# Valorization of European Cranberry Bush (*Viburnum opulus* L.) Berry Pomace Extracts Isolated with Pressurized Ethanol and Water by Assessing Their Phytochemical Composition, Antioxidant, and Antiproliferative Activities

**DOI:** 10.3390/foods9101413

**Published:** 2020-10-06

**Authors:** Lijana Dienaitė, Milda Pukalskienė, Carolina V. Pereira, Ana A. Matias, Petras Rimantas Venskutonis

**Affiliations:** 1 Department of Food Science and Technology, Kaunas University of Technology, Radvilėnų pl. 19, Kaunas LT-50254, Lithuania; lijana.dienaite@ktu.lt (L.D.); milda.pukalskiene@ktu.lt (M.P.); 2 IBET—Instituto de Biologia Experimental e Tecnológica, Food & Health Division Apartado 12, 2780-901 Oeiras, Portugal; carolina.pereira@ibet.pt (C.V.P.); amatias@ibet.pt (A.A.M.)

**Keywords:** guelder rose berries, pomace, pressurized liquid extraction, antioxidant capacity, antiproliferative activity, flavonoids

## Abstract

Defatted by supercritical CO_2_, *Viburnum opulus* berry pomace (VOP) was subjected to consecutive extraction with pressurized ethanol (E) and water (W) and yielded 23% of VOP-E and 8% of VOP-W, respectively. The major phytochemical groups covering 42 identified and quantified constituents in VOP extracts were organic and phenolic acids, iridoids, quercetin and (epi)catechin derivatives, flavalignans, procyanidins, and anthocyanins. The on-line HPLC-DPPH^•^-scavenging assay revealed the presence of numerous antioxidants. VOP-E had a higher total phenolic content, was a stronger antioxidant (equivalent to 0.77, 0.42, and 0.17 g trolox/g in oxygen radical absorbance capacity (ORAC), ABTS, and DPPH assays, respectively), and recovered the major part of phenolics from the pomace; however, both extracts demonstrated similar antioxidant activity in the cellular assay. VOP-E inhibited HT29 cancer cells at non-cytotoxic concentrations. The results of this study revealed that VOP contains valuable phytochemicals possessing antioxidant and antiproliferative activities. Consequently, extracts from VOP substances may be of interest in developing functional ingredients for healthy foods, nutraceuticals, and cosmeceuticals.

## 1. Introduction

Many berry varieties are well-known fruits and are consumed as fresh foods or used as a raw material for various processed products such as jams, juices, beverages, and others. In addition to systematically studied, comprehensively valorized, and widely commercialized berries, there are many underutilized species, which produce the fruits with specific, rather unusual sensory quality and are therefore less acceptable by the consumers as fresh commodities. However, more recently, some of the underutilized berry species have attracted an increasing interest as promising sources of health beneficial bioactive compounds, which, after comprehensive evaluation and systematic valorization, might find wider applications in the production of both regular and, particularly, rapidly developing functional foods, nutraceuticals, and cosmeceuticals [1,2,3,4,5].

European cranberry bush (*Viburnum opulus* L.) berries, also called guelder rose berries, may be assigned to the underutilized fruits and emerging source of food grade products and ingredients [1]. The *Viburnum* genus contains 160–170 species, while guelder rose classified in the subsection *Opulus* contains five species. Many *Viburnum* spp. have become popular as garden or landscape plants, while some of them produce edible red colored fruits with specific astringent taste and unique, although not very pleasant, odor [2]. Some of the sweet cultivars may be consumed as fresh fruits; however, most of the guelder rose berries are mixed with other berries and processed into jams and juice [1]. Some berry phenolic compounds are responsible for organoleptic characteristics such as color and flavor; however, their health promoting properties have been in the focus of numerous studies during the last decades. The major class of phenolic compounds in *V. opulus* is represented by flavonoids including catechins, quercetin glycosides [1,3], proanthocyanidins [4], and phenolic acids [3]. Chlorogenic acid is present as the main phenolic acid [3], while ascorbic acid is another important guelder rose berry bioactive compound [5].

Beneficial effects of dietary phytochemicals have been reported in numerous studies. For instance, the diets rich in flavonoids and other phenolic compounds possessing antioxidant and anti-inflammatory activities have been associated with the reduced risk of various factors associated with metabolic syndrome [6], cancer [7], macular degeneration and eye-related diseases [8], as well as obesity and diabetes [9]. Therefore berry-rich diets are considered as effective means in disease prevention and even their treatment. Earlier studies on *V. opulus* reported its antioxidant activity [5] and strong radical scavenging capacity, mainly due to a high phenolic concentration [10], as well as anticancer activity [11,12,13]. For instance, Ceylan et al. [14] reported that the extracts of *V. opulus* fruits exhibit in vitro and in vivo anticancer activity against Ehrlich ascites carcinoma (EAC) bearing mice, and these effects were related to alkaloids, phenolic compounds, flavonoids, and/or their synergistic effects. Ulger with co-authors [13] demonstrated that the total number of tumor lesions were reduced in mice with 1,2-dimethylhydrazine-induced colon cancer when treated with drinking water enriched with guelder rose juice at the initiation stage of carcinogenesis. Zakłos-Szyda and Pawlik [11] reported anticancer properties of methanol and acetone extracts from the pomace, the juice, and polyphenolic fraction of *V. opulus* fruits in human breast (MCF-7) and cervical (HeLa) cancer cell lines. They suggested that chlorogenic acid, catechin, epicatechin, rutin, quercetin flavonoids, procyanidin B2, procyanidin trimer, and proanthocyanidin dimer monoglycoside present in the obtained preparations may contribute to anticancer activity. Koparal [12] revealed anticancer properties of guelder rose juice, which inhibited Caco-2 and HeLa cancer cell lines but did not have a significant effect in normal and A549 cancer cell lines; anticancer activity was related to the bioactive compounds such as chlorogenic acid and anthocyanins.

Sustainability and waste reduction are among the most challenging issues in fruit processing. For instance, winemaking or pressing juice generates 10–35% of berry pomace, consisting mainly of skins, seeds, and pulp residues. Currently large amounts of berry pomace are discarded as a waste or inefficiently used as a by-product for composting and animal feeding [15]. Considering that polyphenols are distributed in berries unevenly, i.e., approx. 10% of the total polyphenols are present in pulp, 28–35% in skin, and 60–70% in seeds [16], a larger fraction of these bioactive compounds is lost with the pomace after pressing the juice. Therefore, valorization of berry pomace has become a topical issue of research [15]. For instance, the application of high pressure fractionation of dried guelder rose berries and its pomace gave several valuable fractions, namely, polyunsaturated fatty acid rich lipids, higher polarity polyphenolic, and insoluble dietary fiber products [17]. Consequently, berry pomace containing valuable nutrients and being exposed to minimal pesticide levels have been recognized as an attractive source of new food-grade ingredients.

Dried and ground pomace powder can be used directly as food ingredient or processed into higher added value bioactive phytochemical preparations for functional foods, nutraceuticals, and other applications. In the latter case, green and preferably fast extraction techniques have become among the most important research trends for producing higher quality products (extracts) for wider and safer uses [18]. Supercritical fluid extraction with CO_2_ (SFE-CO_2_) and pressurized liquid extraction (PLE) with environment friendly solvents, usually ethanol and water, have been widely used for biorefining berry pomace during last few years [15].

So far, as development of health beneficial ingredients is one of the most important tasks in valorizing berry pomace, comprehensive evaluation of their bioactivities, including antioxidant potential, is a very important issue. Bioactivities related to the antioxidant properties are usually measured by extracellular and cellular methods. A wide range of the in vitro assays have been proposed for this purpose and therefore their selection should be well justified. Determination of DPPH^•^/ABTS^•+^-scavenging and oxygen radical absorbance capacity (ORAC) have been frequently used chemical extracellular assays, while the cellular antioxidant activity (CAA) assay was more recently introduced. Chemical methods, although not always correlating with the in vivo results, are useful for fast screening [19], while CAA is more relevant to the processes occurring in vivo [20]. It involves the studies of phenolic compounds nature, their absorption, distribution, metabolism, and synergistic effect between plant-derived secondary metabolites.

Previous studies demonstrated that berry processing by-products consisting of fruit skin, pulp, and seeds contain high amounts of dietary fiber, vitamins, minerals, phytochemicals, and antioxidants, i.e., natural nutrients, which may play an important role in preventing various degenerative diseases and improving human wellbeing [15]. For instance, there is an increasing evidence supporting the hypothesis that dietary antioxidants may assist the endogenous antioxidative system of protection against oxidative stress by mitigating damaging effects of excessive reactive oxygen species, which may form due to the adverse impact of environmental pollution, inflammatory processes, and other factors.

Considering the increasing interest in guelder rose berry cultivation and processing and still limited studies on biochemical composition and bioactivities of its by-products, this study aimed at a more systematic valorization of bioactive ingredients recovered from berry pomace by PLE with green solvents, ethanol, and water. The following characteristics were determined: (1) total phenolic and anthocyanin content; (2) DPPH^•^/ABTS^•+^-scavenging and ORAC; (3) composition of phytochemicals and their radical scavenging capacities by HPLC-DPPH^•^-scavenging on-line; (4) CAA and cytotoxicity in Caco-2 cells; (5) cell growth activity in a panel of human cancer cells HT29.

## 2. Materials and Methods

### 2.1. Chemicals and Cells

Folin–Ciocalteu reagent, Trolox, DPPH^•^ (98%), gallic acid, KH_2_PO_4_, KCl, NaCl, formic acid (98%), ABTS, (98%), K_2_S_2_O_8_, ammonium hydroxide, AAPH, HPLC grade and LS-MS grade acetonitrile, and HPLC grade formic acid (98%) were obtained from Sigma-Aldrich (Darmstadt, Germany). Disodium fluorescein, Na_2_HPO_4_·2H_2_O, and ethanol (99.9%) were from TCI Europe (Antwerp, Belgium), Riedel-de-Haen (Seelze, Germany), and Scharlau (Barcelona, Spain), respectively. Na_2_CO_3_, 2′,7′-dichlorofluorescin diacetate (DCFH-DA), and quercetin (95%) were from Sigma-Aldrich (St. Quentin Fallavier, France). Ultra-pure water was produced in a Simplicity 185 system (Millipore, Billerica, MA, USA). Analytical grade methanol was purchased from StanLab (Lublin, Poland). The standards used for UPLC analysis (malic acid, fructose, glucose, sucrose, quinic acid, rutin, citric acid, chlorogenic acid) were from Supelco Analytical (Bellefonte, PA, USA); catechin, proanthocyanidin B2, and quercetin-3-O-glucoside were from Extrasynthese (Genay Cedex, France).

Human Caco-2 and HT29 cell lines were purchased from DSMZ (Braunschweig, Germany) and ATCC (Manassas, VA, USA), respectively. The cell culture medium and supplements were purchased from Invitrogen (Gibco, Paisley, UK). Phosphate-buffered saline was obtained from Sigma-Aldrich (St. Louis, MO, USA) and cell viability was assessed using a CellTiter 96^®^ AQueous One Solution Cell Proliferation Assay (Promega, Madison, WI, USA).

### 2.2. Plant Material and Extraction Procedure

Dried *Viburnum opulus* berry pomace (VOP) was kindly donated by BestBerry (Auce, Latvia). According to the information provided by the company, mature berries were pressed in order to remove the juice within 4 h after harvesting; the residue was immediately subjected to drying. Similar preparatory and extraction procedures were applied for VOP as previously reported [17,21]. Briefly, dry VOP was grounded in a laboratory mill Vitek (An-Der, Austria) by using a 0.5 mm size sieve. VOP powder was extracted by SFE-CO_2_ in a 500 mL extractor (Applied Separations, Allentown, PA, USA) to remove lipophilic fraction. For PLE, 10 g of defatted pomace powder was mixed with 4 g of diatomaceous earth, placed in 66 mL extraction cells, and consecutively extracted with ethanol (VOP-E) and water (VOP-W) in an accelerated solvent extraction apparatus ASE350 (Dionex, Sunnyvale, CA, USA) at a constant 10.3 MPa pressure and temperature (70 °C for VOP-E and 120 °C for VOP-W) using 15 min static and a 90 s purge time for each extraction cycle (total 3 cycles, in one run, 120 mL H_2_O and EtOH were used). Ethanol was evaporated in a Rotavapor R-114 (Büchi, Flawil, Switzerland), while residual water was removed by freeze-drying in a Maxi Dry Lyo (Hetto-Holton AIS, Allerod, Denmark). The extracts were weighed and stored at −18 °C in a freezer until further analysis.

### 2.3. Proximate Analysis

The chemical composition of VOP was determined according to the procedures established by the Association of Official Analytical Chemists (AOAC, 1990) [22]: moisture by drying at 105 °C to constant weight; ash by mineralizing in a muffle furnace F-A1730 (Thermolyne Corp., Dubuque, IA, USA) at 500 °C for 3 h; proteins by the Kjeldahl method in a nitrogen analyzer (Leco Instruments Ltd., Mississauga, ON, Canada) using a conversion factor of 6.25; crude lipids by Soxhlet extraction with hexane for 6 h. The rest of the dry matter was assigned to carbohydrates. Each determination was carried out in triplicate.

### 2.4. Total Phenolic Content (TPC) and Antioxidant Capacity

Folin–Ciocalteau (TPC), DPPH, ABTS, and ORAC assays were used for evaluating antioxidant potential of VOP extracts [22]. TPC was performed as described by Singleton and Rossi (1965) with some modifications [23]. A total of 30 μL of extract solutions was mixed with 150 μL Folin–Ciocalteu reagent (1:10 in distilled water) with 120 μL 7% Na_2_CO_3_ solution in a 96-well microplate and the absorbance was measured at 765 nm after 30 min in a FLUOstar Omega Reader (BMG Labtech, Offenburg, Germany). Gallic acid solutions (10–250 μg/mL) were used for the calibration curve. TPC was expressed in mg of gallic acid equivalents in g of extract and pomace, GAE/g DWE (dry weight of extract) and DWP (dry weight of pomace), respectively. 

Re et al.’s (1999) method with some modifications was used for ABTS^•+^ decolourisation assay [24]. A total of 6 µL of sample was added to 294 µL of ABTS^•+^ working solution. The ABTS^•+^ solution was prepared by mixing 50 mL of ABTS (2 mM) with 200 µL of potassium persulfate (70 mM); the mixture was kept in the dark for 15–16 h before use. The working solution was prepared by diluting with PBS (prepared from 8.18 g of NaCl, 0.27 g of KH_2_PO_4_, 1.78 g of Na_2_HPO_4_ × 2H_2_O, and 0.15 g of KCl in 1 L of distilled water) to obtain the absorbance of 0.800 ± 0.030 at 734 nm. The absorbance was measured in a 96-well microplate using a FLUOstar Omega Reader during 30 min at 734 nm. A series of Trolox solutions (399–1198 μM/L) were used for calibration. The results were expressed as µM TE/g DWE and DWP.

Brand-Williams et al.’s (1995) method was applied for the DPPH^•^-scavenging assay with some modifications [25]. A total of 8 μL of sample was mixed with 292 µL of DPPH^•^ methanolic solution (0.0059 g/250 mL). The working solution was prepared by diluting with methanol to obtain the absorbance of 0.7 at 515 nm. After mixing, the microplate was placed in a reader, shaken for 30 s, incubated for 60 min, and the absorbance read at 515 nm. The results were calculated as in the ABTS assay. 

ORAC assay was performed by using fluorescein as a fluorescent probe and AAPH as a peroxyl radical generator [26]. A total of 25 µL of sample was pipetted into 150 µL (14 μM) fluorescein solution, and after incubation during 15 min at 37 °C, 25 µL of AAPH (240 mM) were added. The fluorescence was recorded every cycle (in total, 120 cycles) using 485 excitation and 530 emission fluorescence filters. Antioxidant curves (fluorescence versus time) were first normalized, and from the normalized curves, the net area under the fluorescein decay curve (AUC) was calculated by the following formula: AUC = (1 + f_1_ f_0_ + f_2_ f_0_… fi f_0_) × CT (1), where f_0_ is the initial fluorescence reading at 0 min, f_i_ is the fluorescence reading at time i, and CT is cycle time in minutes. The final ORAC values were calculated by using the regression equation between the Trolox concentration and the net AUC. Trolox solutions (0–250 μM) were used for the calibration. The results were expressed as µM TE/g DWE and DWP. Each measurement was carried out in six replicates.

### 2.5. HPLC-DPPH^•^-Scavenging On-Line

HPLC-DPPH^•^-scavenging online analysis was carried out on a Waters HPLC system (Waters Corporation, Milford, MA, USA) as previously described [21] with minor changes. The Hypersil C18 analytical column (250 × 0.46 cm, 5 µm; Supelco Analytical, Bellefonte, PA, USA) was used for compound separation. The mobile phase consisted of 0.4% formic acid in water (A) and acetonitrile (B). The elution profile was at the start point 90% A, then changing to 60% in 45 min, after that, in 50 min A was decreased to 5%, then in 53 min increased to 90% A, then it was held at 90% for 3 min, and in 2 min it was returned to initial conditions and the column was equilibrated for 5 min. The decrease of absorbance after the reaction of radical scavengers with DPPH^•^ was measured at 515 nm with the variable-wavelength Shimadzu SPD–20A UV detector (Shimadzu Corporation, Kyoto, Japan), while the identification of compounds was performed by using the Waters Acquity UPLC system (Milford, MA, USA).

### 2.6. UPLC-ESI-QTOF-MS Analysis

Quantitative and qualitative analysis was performed with an ultra-performance liquid chromatograph (Waters Acquity UPLC system, Milford, MA, USA) equipped with a quadrupole time-of-flight tandem mass spectrometer (MaXis 4G Q-TOF-MS). Data acquisition and instrument control were performed by using HyStar 3.2 (SR2 software, Bruker Daltonics, Bremen, Germany) [21]. Briefly, samples were eluted with a gradient of solvent A (1% formic acid in ultrapure water) and B (acetonitrile) on a 1.7 µm, 100 mm × 2.1 mm i.d. Acquity BEH C18 column (Waters) over 14 min at a flow rate of 0.4 mL/ min. The injection volume was 1 µL and column temperature was maintained at 40 °C. Gradient elution was performed as follows: 95% A in 0–4 min, 95–90% A in 4–6 min, 90–70% A in 6–10 min, 70–5% A in 10–12 min, and 5–95% A in 12–14 min. The Q-TOF mass spectrometer was equipped with an electrospray ionization (ESI) source in a negative-ion mode. ESI-Q-TOF-MS spectra were recorded in the mass range of 80–1200 *m/z*. Two scan events were applied, namely, full-scan analysis followed by data-dependent MS/MS of the most intense ions. The data-dependent MS/MS used 20–30.0 eV as collision energies (fragmentation was performed by using collision induced dissociation (CID)); the capillary voltage was +4000 V; end plate offset 500 kV; flow rate of drying (N_2_) gas 10.0 L/min; nebulizer pressure 2.5 bar. The same column was used for sugars, while separation was performed using 75% acetonitrile, 25% water, and 0.1% ammonia hydroxide as mobile phase at isocratic conditions at 0.35 mL/min, 10 min, and 35 °C. The method for anthocyanins is described elsewhere [27]. The quantification was performed using cyanidin-3-glucoside as an external standard. The amounts of individual identified compounds were expressed as mg/100 g DWP. A quantitative analysis of other compounds was performed by using calibration curves (0.1–10 µg/mL) of available standards (malic acid, rutin, quinic acid, citric acid, catechin, proanthocyanidin B2, chlorogenic acid, quercetin-3-*O*-glucoside; for sugars glucose, fructose and sucrose were used). Concentrations of phytochemicals were determined from the peak areas, using the linear regression equations of calibration curves obtained from six concentrations of each standard. Quantification trace for quantified compounds were detected by calculating limit of detection (LOD) and limit of quantification (LOQ) values [28]. The results were expressed as mg/100 g DWE and g/100 g DWP.

### 2.7. Cell Culture and Sample Preparation

Water and ethanol VOP extracts were solubilized in DMSO (200 mg/mL) and ethanol (100 mg/mL), respectively, and stored at −20 °C protected from light. Cell-based assays were performed using a maximum concentration of solvents, 1% and 5% for DMSO and ethanol, respectively. Cell lines were cultured in RPMI-1640 medium supplemented with 10% of heat-inactivated fetal bovine serum (FBS) and 1% penicillin-streptomycin (PS), in the case of Caco-2. Cells were maintained at 37 °C with 5% CO_2_ in a humidified incubator and routinely grown as a monolayer in 75 mL culture flasks.

### 2.8. Cytotoxicity Assay in Caco-2 Cell Monolayer

Cytotoxicity was assessed using confluent and non-differentiated Caco-2 cells as a model of the human intestinal epithelium, as previously described [29]. Briefly, Caco-2 cells were incubated with the samples diluted in the culture medium (RPMI supplemented with 0.5% FBS). Cell viability was assessed using CellTiter 96^®^ AQueous One Solution Cell Proliferation Assay (Promega, Madison, WI, USA) containing the MTS reagent, according to the manufacturer’s instructions. The absorbance was measured at 490 nm using a Spark^®^ 10 M Multimode Microplate Reader (Tecan Trading AG, Männedorf, Switzerland) and cell viability was expressed in terms of percentage of living cells relative to the control. The experiments were performed in triplicate.

### 2.9. Cellular Antioxidant Activity (CAA) Assay

The CAA assay was carried out by the procedure of Wolfe and Liu (2008) [20]. In Caco-2 monolayers, 50 µL of PBS, sample and standard (quercetin, 2.5–20 µM) solution, and 50 µL of DCFH-DA solution (50 µM) was added and incubated (60 min at 37 °C, 5% CO_2_). Afterwards, 100 μL of AAPH (12 mM) solution was added to each well containing PBS/quercetin standards/samples, while 100 µL of PBS was added to the blank wells. Fluorescence kinetics was recorded every 5 min during 60 min by using a Microplate Fluorimeter FLx800 (Bioteck Instruments, VT, USA) (excitation and emission wavelengths of 485 nm and 540 nm, respectively). CAA values were expressed as µM of QE/g of extract of independent experiments performed in triplicates.

### 2.10. Antiproliferation Assay

The antiproliferative effect of VOP and standard compounds was evaluated in HT29 cells, as described elsewhere [30]. Briefly, cells were seeded at a density of 1 × 10^4^ cells/well in 96-well culture plates. After 24 h, cells were incubated with different concentrations of the samples diluted in the culture medium. Cell proliferation was measured after 24 h using the MTS reagent, as mentioned above. Results were expressed in terms of percentage of living cells relatively to the control. Three independent experiments were performed in triplicate.

### 2.11. Statistical Data Handling

All experiments were repeated at least three times and the results obtained are presented as means ± standard deviations (SD). Statistical unpaired *t* test with *p* < 0.05 was used to identify significant differences.

## 3. Results and Discussion

### 3.1. Proximate Composition, Total Yield, and Antioxidant Capacity

Berry pomace are highly heterogeneous materials, which are composed of macronutrients (mainly proteins, fats, and carbohydrates) and a large variety of micronutrient phytochemicals such as phenolic acids, flavonoids, and glycosides. The latter compounds belong to the class of polyphenols and are promising substances for developing natural antioxidants and functional food ingredients with various bioactivities. In this study, a proximate analysis of VOP was performed. The content of protein, fat, water, and ash in VOP was 16.82 ± 1.54%, 26.24 ± 0.59%, 7.76 ± 0.16%, and 1.21 ± 0.03%, respectively. Other macrocomponents should consist of various carbohydrates. For comparison, Polka et al. [31] reported approximately 3-fold and 2.5-fold lower amounts of protein and fat, respectively, and a 2.4-fold higher amount of ash in dried guelder rose berries.

Polyphenolic antioxidants and ascorbic acid, which are abundant in various fruits, are the most valuable constituents of berries, firstly, as health beneficial compounds and, secondly, as natural additives, which in some cases may be used to replace synthetic preservatives and colors. Therefore, the evaluation of an antioxidant potential of VOP was among the main objectives of this study. The antioxidant capacity of extracts (expressed for DWE) and the recovery of antioxidants from the dry VOP (expressed for DWP) were determined for each assay for this purpose. Both characteristics are important for pomace valorization, the former evaluates antioxidant properties of the product (extract, fraction), while the latter evaluates the efficiency of the extraction process. For instance, a low yield extract may possess stronger antioxidant activity, while in the case of high yields, better recovery of antioxidants from the plant may be achieved, although the extracts may show lower antioxidant capacity due to the dilution of active constituents with the neutral ones. An extraction with ethanol resulted in an approximately three times higher total extract yield comparing to water (Table 1); furthermore, antioxidant capacity values of VOP-E were significantly higher than those of VOP-W in the all assays. 

In addition, ethanol, due to remarkably higher yield, recovered a 3.4–3.8 times higher amount of polyphenolics from the pomace (DWP). This could be explained by the higher content and wider spectra of various soluble components in ethanol during the first step of PLE (Table 2). Despite the differences in antioxidant capacity values determined by the applied methods, which may be explained by different chemical structures of used radicals for the assay and the peculiarities of reaction mechanisms, the scavenging capacity and TPC of VOP-W were also quite high.

Most likely, the increased temperature in PLE with water promotes the recovery of polyphenols from the used residue after extraction with ethanol due to the breakdown of the cell walls and increase of membrane permeability. In addition, heating might soften plant tissue and weaken the interactions between phenols and biopolymers present in the raw material, namely, proteins and polysaccharides. Consequently, the diffusion of phenolics into the solvent would increase.

Some data on antioxidant capacity of guelder rose berry juice and pomace extracts are available from the previously performed studies. Significantly lower TPC values were determined in different juice samples (5.47–10.61 mg GAE/g) while the antioxidant capacity varied from 127.37 to 260.38 and from 31.95 to 109.81 µmol TE/g in the ORAC and ABTS assays, respectively [1]. Zakłos-Szyda et al. [11] produced a polyphenol-rich fraction from the guelder rose berries by solid phase extraction under pressure; it contained 361.52 mg GAE/g compared to 5.98 mg GAE/g in juice. TPC values of methanol and acetone extracts of VOP reported in the aforementioned study agreed with our results. Kraujalis et al. [17] reported TPC in different VOP extracts from 88.6 to 74.9 mg GAE/g DW and it was higher in the water extract. The same tendencies were observed for the antioxidant capacity determined by the DPPH, ABTS, and ORAC methods. Comparing with similar products obtained from sea buckthorn berry pomace [21], guelder rose berry pomace extracts were remarkably stronger antioxidants in the all applied assays and exhibited larger TPC values. It may be also noted that the concentration of secondary metabolites as well as some macronutrients is often larger in the berry pomace compared to the fruit pulp [11,31].

### 3.2. Characterization and Quantification of Phenolic Compounds in VOP Extracts

**Figure 1:**
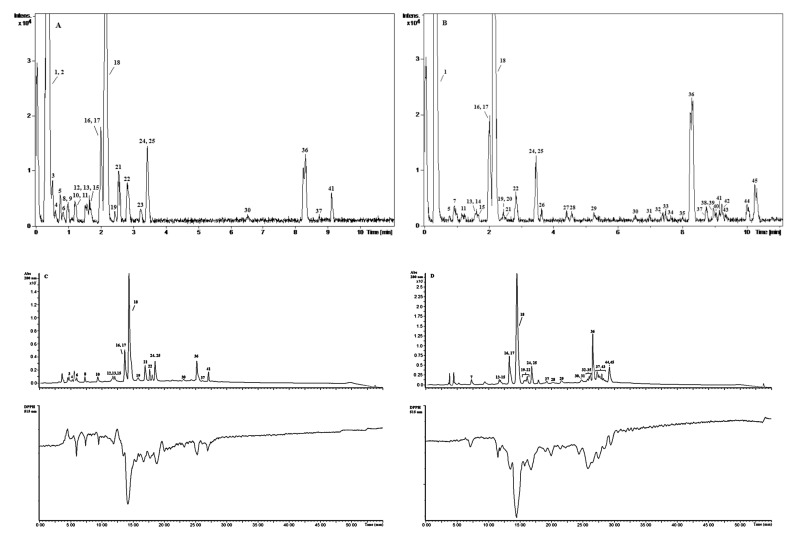
Representative UPLC-QTOF-MS chromatograms of water (**A**) and ethanol (**B**) extracts of VOP. HPLC-UV-DPPH^•^-scavenging chromatograms of water (**C**) and ethanol (**D**) extracts of VOP.

The identification and quantification of phenolic acids and flavonoids in the extracts was of major interest for valorizing their potential to provide health benefits. It is well-known that the type and the structural peculiarities of phenolics may predestine extract’s antioxidant and other activities. The composition of VOP extracts was analyzed by UPLC-QTOF-MS, while the radical scavenging capacity of the separated compounds was additionally monitored by the HPLC-DPPH^•^-scavenging on-line method. As a result, 45 phytochemical compounds were detected of which 42 were identified and 45 quantified using calibration curves produced with various analytical standards. It may be observed that most of them belong to flavonoids (Figure 1A,B, Table 2). Combined UV (positive signals) and DPPH^•^ quenching (negative signals) chromatograms of VOP extracts isolated from SFE-CO_2_ residue are presented in Figure 1(C: VOP-W; D: VOP-E). In the latter assay, the HPLC-separated antioxidants react post-column with DPPH^•^, the solution of which is circulating in the coil. The induced bleaching of a radical is detected as a negative peak photometrically at 515 nm. Phenolic acids (**3**, **6**, **8**, **10**, **12**, **18**, **21**, **25**, **35**), iridoids (**7**, **39**, **42**–**45**), flavalignans (**28**, **31**, **36**, **37**, **41**), (epi)catechin derivatives (**4**, **13**, **15**, **16**, **19**, **20**, **22**, **24**, **27**, **34**, **40**), quercetin derivatives (**30**, **32**, **33**, **38**), and other flavonoids (**14**, **17**, **29**) were found to be active radical scavengers in the investigated extracts. The VOP-E extract was a stronger radical scavenger than the VOP-W extract. The activity of VOP-W was comparable with that of phenolic acids, especially chlorogenic and hydroxybenzoic, and catechin derivatives (**13, 15, 16, 22, 24**), while the activity of VOP-E was considerably higher, most likely due to the presence of other constituents such as iridoids (**7**, **39**, **42**–**45**), flavalignans (**28**, **31**), quercetin derivatives (**33**, **38**), and other flavonoids (**14**, **29**, **32**, **35**). The compounds **6**, **8**, **10**, **12**, **16**, **18**, **24**, **25**, **36**, **41** and **7**, **13**, **14**, **16**, **18**, **20**, **24**, **25**, **28**, **30**, **33**, **36**, **41**, **42**, and **45** in VOP-W and VOP-E, respectively, were detected as the main antioxidants responsible for extracts antioxidant capacity. Chlorogenic acid was the strongest radical scavenger, while the input of other constituents, as it may be observed from the negative peaks in the chromatograms, was remarkably lower. Chlorogenic acid and rutin were identified based on retention time and MS of authentic standards. When the standards were not available, the identification was performed by comparing the exact mass of the parent ion (M-H) and typical MS fragmentation pattern with available data in previously published articles and PubChem, Chemspider, and Metlin databases.

Malic (**1**), citric (**2**), and quinic (**5**) acids were identified by using standards. Citric acid was detected only in VOP-W. These acids were reported in *V. opulus* previously [1,32,33]. Perova et al. [33] in 100 g of eleven tested guelder rose berry varieties collected from different locations determined 578.0–2090, 279.0–1630, and 52.0–346.0 mg of malic, citric, and quinic acids, respectively. It is not surprising—the major part of soluble organic acids is transferred to the juice during pressing. Based on deprotonated ion *m/z* 371 and a fragment *m/z* 175, corresponding to the glucuronide moiety, compound **3** was tentatively identified as dihydroferulic acid 4-*O*-glucuronide. This compound was identified and quantified for the first time as a minor constituent in VOP-W extract. Compound **4** generated the fragment *m/z* 423 by RDA (retro-Diels-Alder), while other two fragments, *m/z* 467 and 305, were originated due to HRF (heterocyclic ring fusion) and QM (quinone methide cleavage) fragmentation, respectively. The fragment *m/z* 289 was formed due to the cleavage of the interflavan C–C bond corresponding to the (epi)catechin. Therefore, this compound was assigned to the procyanidin derivative. A small amount of this compound was detected only in the VOP-W extract.

Three benzoic acid derivatives were detected in VOP extracts. The compounds **6** and **8** gave similar [M-H]^−^ ion *m/z* 153 and fragment ion *m/z* 109 (the loss of [M–H–CO_2_]^−^), while the compound **12** with parent ion *m/z* 137 gave a fragment *m/z* 93 (the loss of CO_2_), which coincided with the fragmentation pattern of hydroxybenzoic acid derivatives containing two and one hydroxyl groups, respectively. Structurally, hydroxybenzoic acids may contain as many as four hydroxyl groups surrounding a single benzene ring. Hydroxyl groups in natural compounds are usually attached to the 3, 4, and 5 positions which cannot be exactly determined by MS. Therefore, on the basis of the mass spectra and previously published data, the peaks **6**, **8**, and **12** were tentatively identified as di- and mono-hydroxybenzoic acid derivatives [34]. All three compounds were detected and quantified in VOP recovered with water, the amount of **12** was eight-fold lower than reported by Velioglu et al. [3].

**Figure 2:**
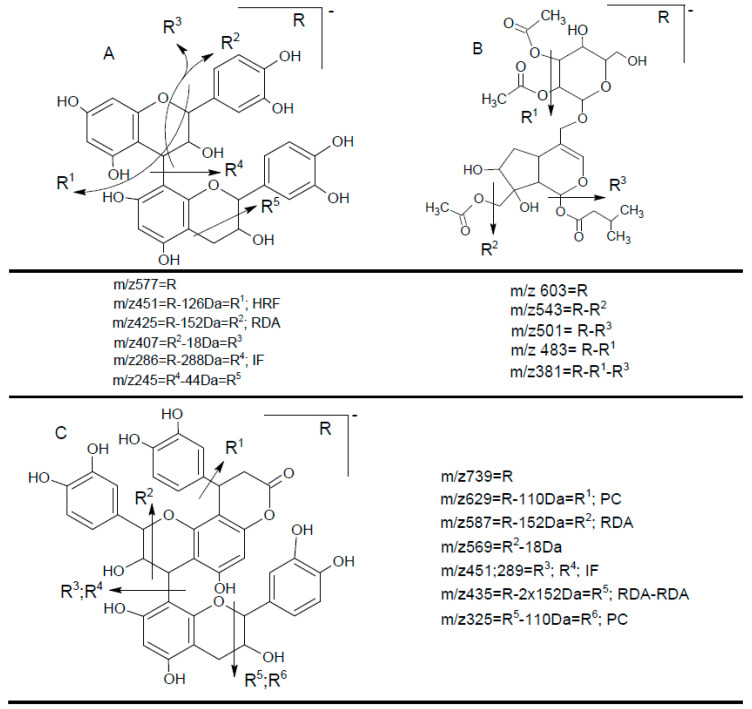
Hypothetic structures of selected daughter ions resulting from MS-MS fragmentation of the compound 13 procyanidin dimer B2 (**A**), 45 opulus iridoid II (**B**), and 28 cinchonain IIx (**C**). HRF—heterocyclic ring fussion; PC—phenyl cleavage; RDA—retro-Diels-Alder reaction; IF—interflavan fussion.

The peaks **7**, **39**, **42**, **43**, **44**, and **45** were identified as iridoids (Table 3, Figure 2). The compound **39** produced a molecular ion *m/z* 373 [M−H]^−^ in MS spectra and the fragments *m/z* 123, 149, 167, 193, and 211 in the MS^2^ spectra. The fragment ion 211 [M–H–162]^−^ corresponded to the loss of glucose residue, while further loss of H_2_O produced the fragment *m/z* 193. The ion *m/z* 149 could be formed due to the loss of H_2_O and CO_2_ from *m/z* 167 and *m/z* 193, respectively. While the ion *m/z* 123 could originate from *m/z* 149 and *m/z* 167 by the loss of C_2_H_2_ and CO_2_, respectively. As compared with the MS fragments and literature, peak **39** was plausibly characterized as secologanate [35]. This compound was not reported in *V. opulus* previously. Peak **7** gave pseudomolecular ion *m/z* 441 and several fragment ions listed in Table 2. The fragmentation pathway was similar to iridoids via formation of acetic and valeric acid moieties; however, MS data was not sufficient to elucidate the exact structure of this iridoid.

Other compounds, based on the previously reported in *V. opulus* fruits data and fragmentation patterns, were assigned to viburtinoside and opulus iridoids [32,36]. For example, MS^2^ fragments of viburtinoside derivatives (42, 43) indicate the sequential neutral losses of two acetic and one (iso)valeric acid groups, while opulus iridoid III (44) had three acetic and one (iso)valeric acid moieties. Opulus III (45) contains additional pentose moiety. Bock et al. [37] and Bujor et al. [32] reported the detailed fragmentation pathways for these compounds. In our study, iridoids constituted one of the major parts of all quantified phytochemicals, and they were recovered only in ethanol. According to the recovered amount, their content in 100 g of dried VOP was from 1.21 ± 0.01 mg (43) to 10.51 ± 0.53 mg (45). These constituents were quantified for the first time in VOP.

Peak **9** was detected only in VOP-W and exhibited the molecular ion *m/z* 161 and fragment ions *m/z* 143 (–18 Da) and 133 (–28 Da), which suggested a dimethyl ester derivative of malic acid. Four derivatives (**10**, **18**, **21**, and **25**) of chlorogenic acid were detected in VOP (Table 3, Figure 2). An interpretation of their MS^2^ spectra using previously developed hierarchical keys enabled to assign these peaks to a particular positional derivative [38,39,40]. Furthermore, it was found that the fragment ion type and ion intensity were in a good correlation with the position of the caffeoyl group. The characteristic fragments *m/z* 133, 135, 161, 173, 179, and 191 were formed due to the cleavage of an ester bond between caffeoyl and quinic acid groups, quinic acid dehydration, along with caffeoyl decarboxylation. The compounds **10** and **25** shared a similar fragmentation pattern (Table 2); these molecules produced a base ion *m/z* 191 ([quinic acid −H]^−^) by the loss of a caffeoyl residue, an ion *m/z* 161 ([caffeoyl–H−H_2_O]^−^) by the loss of C_7_H_12_O_6_, another ion *m/z* 135 ([caffeoyl–H−CO_2_]^−^) by the loss of C_8_H_10_O_7_, and finally one more ion *m/z* 133 ([caffeoyl–H−H_2_O−CO]^−^).

The absence or low intensity of *m/z* 179 ([caffeoyl−H]^−^) enabled to separate them from 3-O-caffeoylquinic acid and to identify as 1- and 5-caffeoylquinic acids [40]. Thus, compound **10** was identified as 1-caffeoylquinic acid, while the absence of *m/z* 179 for peak **25** let us to identify it as neochlorogenic acid. Compound **18** was identified as 3-O-caffeoylquinic acid (chlorogenic acid); it yielded the base peak *m/z* 191 (deprotonated quinic acid) and also gave an ion *m/z* 179 [M–caffeic acid–H]^–^ with half of the intensity of the base peak. Moreover, it was confirmed by the analytical standard. Compound **21** gave characteristic ion *m/z* 173 [M–quinic acid–H–H_2_O]^–^ and was identified as 4-O-caffeoylquinic acid (cryptochlorogenic acid) according to the previously described fragmentation pattern [39,40]. Chlorogenic acid, as the main phenolic compound in fresh berries of *V. opulus*, has been reported previously [1,3,33]. Chlorogenic acid and its derivatives are among the major constituents in VOP extracts as well. The content of these phenolic acids recovered from 100 g of VOP varied from 0.49 ± 0.00 to 214.8 ± 0.02 mg. Chlorogenic acid (**18**) was dominant in VOP-W, while 1-caffeoylquinic acid (**10**) was detected and quantified only in VOP-W. For comparison, the content of chlorogenic acid was similar to the previously reported in guelder rose berry juice by Velioglu et al. [3]; however, 1.16–2.7 times lower than reported by Perova et al. [33] in guelder rose fruits.

MS data for compounds **11** and **26** were not sufficient for their identification. Despite that, these compounds were quantified. Moreover, spectral information of these compounds are presented in the Supplementary Materials; the content of **11** was 52-fold higher in VOP-E, than in VOP-W, while compound **26** was determined and quantified only in VOP-E. 

The compounds **13**, **20**, **22**, and **34** gave *m/z* 577 corresponding to a molecular ion [M–H]^–^ of a procyanidin dimer with the MS fragments *m/z* 451, 425, 407, 289, and 245 (Figure 2). The fragment *m/z* 425 is a result of RDA (retro-Diels–Alder) fission of flavonoid nucleus, which gives rise to *m/z* 407 after the loss of H_2_O. An ion *m/z* 451 indicates the loss of the A-ring (126 Da) [41]. The cleavage of the interflavan C–C bond produced *m/z* 289 corresponding to the catechin (epicatechin) unit, while further loss of 44 amu (C_2_H_4_O) in the benzofuran skeleton yielded *m/z* 245. Consequently, the extract had some procyanidin dimers, which have different retention times due to different bonding of catechin and epicatechin moieties. In addition, compound **13** was confirmed by the authentic standard as proanthocyanidin B2. The content of recovered procyanidin dimers varied from 0.48 ± 0.00 to 8.89 ± 0.01 mg/100 g DWP. The recovered content of **13** and **22** was similar by both solvents, while the compounds **20** and **34** were found only in VOP-E with an approximately five-fold higher content of **20** than **34**. Procyanidin dimers were reported previously without quantification [1,32,41]; the concentration of procyanidin dimer determined in guelder rose berry juice [3] was similar to its content in VOP determined in our study.

The deprotonated ion [M-H]^–^ *m/z* 515 of **14**, as well as MS^2^ fragments *m/z* 353 [M–scopoletin-hexose–H]^–^, 341 [M–sophoroside–H]^–^, 191 [scopoletin-H]^–^, and 179 [M–hexose–H]^–^ indicate on the structure of scopoletin-7-sophoroside [42]. To best of our knowledge, this compound was not previously reported in VOP (Table 3). This compound was identified and quantified only in VOP-E, which recovered 1.96 ± 0.11 mg/100 g DWP.

Compound **15** was tentatively assigned to (epi)catechin-dihexoside on the basis of its fragment ions *m/z* 271, 289, 451, and 503. The loss of the B ring (110 amu) from the parent ion could explains the fragment *m/z* 503. The sequential loss of two hexosyl moieties from *m/z* 613 produced *m/z* 451 [M–hexosyl–H]^–^ and 289 [M–2×hexosyl–H]^–^. Elimination of H_2_O from aglycone generated the fragment *m/z* 271. This compound was not identified previously in *V. opulus*. Moreover, compound **19** has one sugar moiety less than compound **15**. Previously, this compound was identified in *V. opulus* fruits [32]. The typical (epi)catechin aglycone (*m/z* 289) fragmentation was proved by the MS^2^ spectra by showing the loss of 162 Da, and further loss of 44 Da (C_2_H_4_O) in the benzofuran skeleton yielding *m/z* 245. Finally, loss of the B ring (110 amu) from the parent ion yielded an ion *m/z* 341. The recovered content of **19** was similarly recovered by both solvents, while the content of compound **15** was eight-fold higher in VOP-W. These compounds were reported previously without their quantification [32,41].

The compounds **16** and **24** eluting at 2.0 and 3.4 min, respectively, displayed the same molecular ion *m/z* 289 (C_15_H_13_O_6_). Furthermore, they gave the same characteristic MS^2^ fragments *m/z* 245 (loss of CO_2_), 205, and 203 (cleavage of the A-ring of flavan-3-ol). The identity of compound **16** was finally proved using the catechin analytical standard. The hydrophobicity of epicatechin is higher than catechin; therefore, **24** eluted later than **16** and was assigned to epicatechin [32,43]. The contents of catechin and epicatechin in *V. opulus* berry juice were 29.04 and 2.69 mg/100 g, respectively. Comparing to Velioglu et al.’s [3] study, ethanol recovered higher contents of catechin, while water produced lower amounts. Recovered epicatechin values were three- to six-fold higher than reported by Velioglu et al. [3].

The compounds **17** and **23** yielded molecular ion *m/z* 579, but exactly suggested two different molecular formulas: C_30_H_28_O_12_ and C_26_H_28_O_15_, respectively. Compound **17** was tentatively identified as gambiriin based on the characteristic MS^2^ fragmentation pattern consisting of base *m/z* 289, which fragmented into *m/z* 245 and 205 after RDA and QM [44]. To the best of our knowledge, this compound was identified and quantified for the first time in VOP. Compound **23** generated several fragments in negative ionization mode (Table 2); however, this information was not sufficient for its tentative identification. Furthermore, spectral information of this compound are presented in the Supplementary Materials. The latter compound was detected and quantified only in VOP-W, while the calculated concentration of gambiriin was approximately two times higher in VOP-E than in VOP-W.

The precursor ion *m/z* 865 was detected for compound **27**. The fragment *m/z* 739 was caused by HRF reaction of the upper unit, while *m/z* 713 occurred due to a RDA reaction of (epi)catechin unit, while *m/z* 695 indicates the loss of H_2_O. The fragment *m/z* 577 corresponds to the (epi)catechin dimer and originates from a QM reaction. Furthermore, the fragment ion of the dimer was observed following the RDA-based reaction (*m/z* 425), which yielded *m/z* 407 owing to the subsequent loss of H_2_O. The HRF reaction was also observed for the dimer, generating *m/z* 451. Finally, a QM reaction with the dimer formed at single quinone, resulting in *m/z* 289 and 287 ions. Therefore, this compound was tentatively assigned to procyanidin C1 [1,32,41]. This compound was quantified only in VOP-E with the recovery value of 2.20 mg/100 g DWP.

Five flavalignans in the form of flavanols substituted with phenylpropanoids were found in VOP (Table 3, Figure 2). The molecular ion of **31**, **36**, **37**, and **41** with *m/z* 451 (C_24_H_19_O_9_) produced the fragments *m/z* 341, 231, 217, 189, and 177, while the molecular ion of **28** with *m/z* 739 (C_39_H_31_O_15_) typically fragmented to *m/z* 629, 587, 569, 451, 435, 325, and 289. Based on this information, the compounds **31**, **36**, **37**, and **41** were identified as cinchonains Ix (x = a, b, c, or d), while compound **28** was assigned to one of cinchonains IIx (x = a, b). The structures of these compounds together with the fragmentation pathways were proposed by Hokkanen et al. [45]. Purification and NMR would be required for more precise identification of compounds **28**, **31**, **36**, **37**, and **41**. Cinchonains were previously found in lingonberries and bilberries [45], while their presence in *V. opulus* has not been reported. Flavalignans constituted another large group of phytochemicals quantified in VOP; their concentration varied from 0.10 ± 0.00 to 59.84 ± 1.41 mg/100 g DWP. Quantitatively, compound **36** was dominant in both extracts with higher value in VOP-E. The compounds **28** and **31** were quantified only in VOP-E, while the content of **37** and **41** was higher in VOP-E and VOP-W, respectively. 

Quercetin-3-O-glucoside (**32)** and rutin (**33)** were unambiguously identified by the obtained MS data and by using available standards. These compounds were quantified in VOP-E. Both of them were reported previously [3,11,32,33,41]. The amount of **33** quantified in guelder rose berry juice by Velioglu et al. [3] was approximately three-fold lower compared to our study, while the content of **32** determined by Zakłos-Szyda et al. [11] varied from 0.01 to 1.06 mg/g of extract depending on the type of sample preparation.

Compound **29** present in VOP-E gave *m/z* 319 ion (C_15_H_12_O_8_) and, based on the detailed fragmentation pattern reported by Fan et al. [46], was tentatively identified as dihydromyricetin. This compound has not been reported in *V. opulus* previously. The compounds **30** and **38** eluting at 6.5 and 8.9 min with a similar pseudomolecular ion [M–H]^–^ *m/z* 595 were assigned to quercetin pentoside hexoside. The identity is supported by the characteristic fragment ions, especially the diagnostic ion *m/z* 301.0356 of deprotonated quercetin aglycon, the loss of hexose (162 Da) and pentose (132 Da) [32,41]. Compound **38** was quantified only in VOP-E, while the amount of **30** was similar in both extracts.

Compound **35** detected in the VOP-E produced *m/z* 381 (C_18_H_21_O_9_); based on MS, it was tentatively identified as ethylchlorogenate. This compound was identified and quantified for the first time in VOP. The most intense transition used for quantification of the compound **40** was the fragment *m/z* 739, which yielded *m/z* 289 due to the well-known quinone methide cleavage (QM). Fragments ions *m/z* 577, 451, 339, and 177 were formed due to the loss of hexose (162 Da), heterocyclic ring fusion (–287 Da) of ring C of upper unit, the loss of ring B from the upper unit after the 739 ion QM cleavage, and finally, the loss of hexose (162 Da) from the *m/z* 339. Based on this and reported data, compound **40** was tentatively identified as (epi)catechin dimer monoglycoside [41,47]; it was detected and quantified only in VOP-E.

The most important soluble sugars were quantified by using authentic standards: their amounts in 100 g DWP varied from 0.87 ± 0.12 to 2.98 ± 0.51 g of glucose, from 1.09 ± 0.11 to 5.48 ± 1.01 g of fructose, and from 1.17 ± 0.54 to 2.69 ± 0.50 g of sucrose, in VOP-E and VOP-W, respectively. Approximately 4.5–6-fold higher levels of quantified sugars were determined in VOP-W. Perova et al. [33] reported 2.3–4.0 g/100 g fructose and 2.8–4.6 mg/100 g glucose in 11 varieties of guelder rose berries collected from different locations; while sucrose was determined only in three samples in the range of 0.04–0.14 mg/100 g.

In total, three anthocyanins were quantified in VOP-E and one in VOP-W (0.090 ± 0.02 mg/100 g DWP). Cyanidin-3-O-gliucoside (15.18 ± 0.08 mg/100 g DWP) was the dominant anthocyanin in VOP-E followed by cyanidin-3-rutinoside (2.12 ± 0.17 mg/100 g DWP) and cyanidin-3-xylosyl-rutinoside (0.14 ± 0.05 mg/100 g DWP). Perova et al. [33] tested 11 *V. opulus* berry varieties and detected eight anthocyanins. Total anthocyanin amounts were in the range of 11.0–31.0 mg/100 g of fresh berries. The relative content of cyanidin-3-xylosyl-rutinoside was the highest (in some varieties constituted up to 95.7%), followed by cyanidin glucoside (up to 77%) and cyanidin-3-rutinoside (up to 20.6%). Zakłos-Szyda et al. [11] reported anthocyanins in *V. opulus* berry juice and extracts (in mg/g): cyanidin-3-glucoside 0.20 ± 0.01–17.08 ± 0.01, cyanidin-3-rutinoside 0.06 ± 0.0–5.21 ± 0.01, and cyanidin-3-sambubioside 0.85 ± 0.0–55.78 ± 0.69. The highest values were observed in the polyphenol rich fraction (78.06 ± 0.68 mg/g), while the lower ones in juice (1.12 ± 0.0 mg/g). Our results, in general, are in the rage of the reported values. Anthocyanins, as the compounds demonstrating antioxidant, anti-inflammatory, and anti-apoptotic effects, have been proposed for the prevention and treatment of various diseases and disorders [48].

Our study demonstrated that ethanol effectively extracts phenolic compounds; however, PLE with water revealed that residual amounts of various phytochemicals, particularly higher polarity hydrophilic compounds, still remain in the residue after PLE with ethanol. On the other hand, it should be noted that high PLE temperature may increase the risk of decomposition of some thermolabile components, while some of them may undergo partial hydrolysis. In general, the results showed that VOP is rich in a large diversity of polyphenols, most of which were shown to possess strong antioxidant activity. It is known, that hydroxyl position in the molecule and some other structural properties determine antioxidant properties of flavonoids; in general, they depend on the ability to donate hydrogen or electron to a free radical. In addition, the variations in the content of reported compounds could also occur due to genotype, origin, harvesting time, and juice processing parameters.

### 3.3. Antiproliferative Activity and Cytotoxicity Effects of VOP Extracts

In order to evaluate the antiproliferative activity of VOP extracts, HT29 cells were used at the exponential grow phase, while cytotoxicity assessment used Caco-2 cell line as the best accepted intestinal model. When confluent, Caco-2 cells share some characteristics with crypt enterocytes and, therefore, have been a widely implemented cell model to assess the effect of chemicals, food compounds, and nano/microparticles on the intestinal function [49]. Thus, HT-29 and Caco-2 were selected as convenient cell models to evaluate the anticancer effects of VOP extracts.

**Figure 3:**
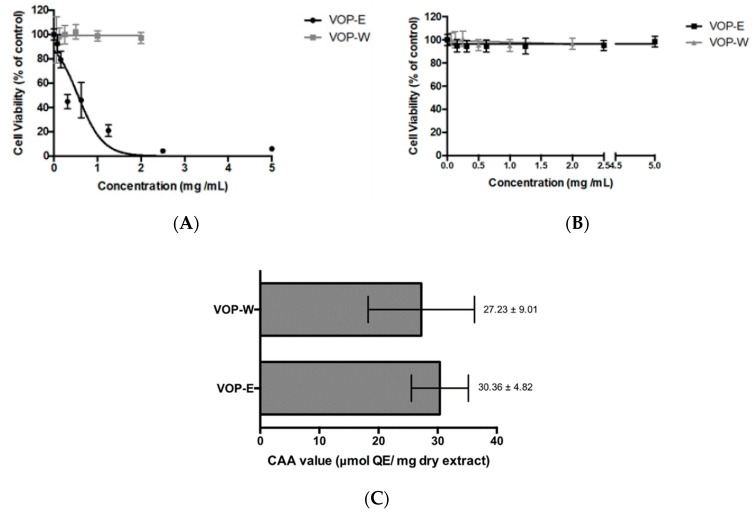
Dose–response curves of *Viburnum opulus* berry pomace (VOP) extracts. Antiproliferative (**A**) and cytotoxicity (**B**) effects using HT29 and Caco-2 cell lines, respectively. Results are expressed in terms of mean ± SD performed in triplicate. Antioxidant activity of VOP extracts evaluated by the cellular antioxidant activity (CAA) assay (**C**). Results are expressed as μmol QE per mg of dry extract of mean ± SD performed in triplicate. Unpaired *t* test was assessed with *p* < 0.05.

To the best of our knowledge, the antiproliferative activity and cytotoxicity of VOP extracts in this study are reported for the first time. Both extracts were diluted in the solvents at their maximum acceptable concentration levels; VOP-E strongly inhibited cancer cell growth with an EC_50_ value of 0.39 ± 0.03 mg/mL (Figure 3A), without adverse effects on normal epithelia once no cytotoxicity was observed on Caco-2 (Figure 3B). VOP-W did not exert an antiproliferative effect at 2 mg/mL concentrations, while higher doses were not applied. The antiproliferative effect of VOP may be attributed to the presence of active phytochemicals and/or their interactions (Table 2). Procyanidins, catechin, epicatechin, hydroxybenzoic acids, anthocyanins, quercetin derivatives, as well as chlorogenic acid and its derivatives strongly suppressed cancer cell growth [11]. VOP-E contained higher amounts of gambiriin (**17**), cinchonains (**36**, **37**), and anthocyanins.

In addition, the antiproliferative effect of VOP-E could be attributed to the presence of other phenolic compounds, which were not found in VOP-W, e.g., cinchonains (**28**, **31**), iridoids (**7**, **39**, **42**–**45**), (epi)catechin derivatives (**20**, **27**, **34**, **40**), quercetin derivatives (**32**, **33**, **38**), scopoletin-7-*O*-sophoroside (**14**), and dihydromyrecitin [50] (**29**) and ethylchlorogenate (**35**). The bioactivities of most of these phytochemicals have not been reported previously. The strong anticancer compound scopoletin [51] is an aglycone of scopoletin-7-*O*-sophoroside (**14**), which might exert anticancer activity after hydrolysis. Moreover, the information on *V. opulus* iridoids and their bioactivities is rather scarce. Some iridoids demonstrated remarkable effects against various cancer cells [52]. It is also worth mentioning that the compounds showing other bioactivities such as antimicrobial and antiviral usually are promising candidates for anticancer agents. For instance, Ming et al. [53] reported antibacterial, antifungal, antiviral, and hepatoprotective properties of cinchonains. In general, VOP-E showed an antiproliferative effect against HT-29 cells in vitro and could be a promising substance for expanding its testing using experimental animal models.

### 3.4. Cellular Antioxidant Activity (CAA) of VOP Extracts

In contrast to antiproliferative and cytoprotective properties, both VOP extracts showed strong and similar antioxidant activity with CAA values of 27.23 ± 9.01 and 30.36 ± 4.82 μmol QE/mg DWE for VOP-W and VOP-E, respectively (Figure 3C). It is interesting noting that despite no antiproliferative activity of VOP-W in HT29 cells its antioxidant activity in Caco-2 cells was similar. It may be assumed that the antiproliferative effects of antioxidant phytochemicals of VOP, due to structural peculiarities, may be achieved by different mechanisms, which are not directly related to their antioxidant capacity measured by the CAA assay. For example, phenolic hydroxyl groups at ortho or para positions with respect to each other increase the antioxidant activity due to additional resonance stabilization and *o*-quinone or *p*-quinone formation [54]. Two *ortho* hydroxyl groups contribute more to the radical-scavenging capacity than do the two hydroxyl groups in *meta* position. For example, protocatechuic acid has two hydroxyl groups in the *ortho* position and possesses higher antioxidant activity than γ-resorcylic acid with two hydroxyls in *meta* position [25]. Furthermore, higher amounts of procyanidin dimers (**13**, **22**), (epi)catechin derivatives (**15**, **19**, **41**), catechin (**16**), epicatechin (**24**), chlorogenic (**18**), and cryptochlorogenic acids (**25**) had influence for high cellular antioxidant activity of the VOP-W extract. Additionally, the extracts demonstrated similar activity in CAA, while VOP-E showed stronger antioxidant capacity in non-cellular chemical-based assays. The CAA assay using a reduced DCFH-DA probe takes into account the bioavailability, distribution, and metabolism of antioxidants within the cell and reflects the activity of the tested extracts in the biological system [55]. It could be related to the structure of bioactive compounds; for instance, flavonoids with 2,3-double bond and 4-oxo group such as quercetin usually possessed high CAA activity [55]. Zakłos-Szyda and co-authors [56] evaluated chemoprotective activity of *V. opulus* fruit juice, acetone and methanol extracts of pomace, and polyphenol-rich fraction in Caco-2 cells using a strong pro-oxidant and in vitro oxidative stress inducer *tert*-butylhydroperoxide (t-BOOH). The extracts and especially the polyphenol-rich fraction strongly protected the cells from oxidative damage. Therefore, it was suggested that an antioxidant activity of extracts depends on the composition of polyphenolic compounds such as catechin, epicatechin, anthocyanins, procyanidin B1 and B2, neo and chlorogenic acid, rutin other quercetin derivatives, and hydroxycinnamic acids. Pre-incubation with guelder rose fruit extract at 0.365 mg/mL also protected βTC3 cells against oxidative damage induced by t-BOOH [57]. Consequently, the extracts of previously studied *V. opulus* fruits and in our study investigated pomace contain valuable bioactive compounds with cytoprotective properties.

## 4. Conclusions

Forty-two bioactive phytochemicals were characterized in defatted guelder rose berry pomace extracts recovered by consecutive extraction with pressurized ethanol and water. Dihydroferulic acid 4-O-glucuronide, scopoletin-7-O-sophoroside, gambiriin, five flavalignans, dihydromyrecitin, and ethylchlorogenate, as well as antiproliferative and antioxidant activity of *V. opulus* berry pomace (VOP) extracts are reported for the first time. Ethanol extract showed higher antiproliferative activity with no cytotoxicity at the applied doses. Both extracts showed strong antioxidant activity in Caco-2 cells protecting them upon stress stimuli. The differences in the antioxidant power and antiproliferative activity of extracts may be related to a phytochemical composition and structural peculiarities of the compounds, particularly to the distribution of hydroxyl groups in the molecules. Considering strong antiproliferative and antioxidant properties, guelder rose berry pomace extracts, especially isolated by ethanol, may find promising applications in functional foods and nutraceuticals. However, technological upscaling and economic issues should be further evaluated in order to assess the possibilities of industrial production of ingredients from *V. opulus* berry pomace.

## Figures and Tables

**Table 1:** Yield, total phenolic content, and antioxidant capacity of guelder rose berry pomace extracts.

Characteristic	Material	VOP-E	VOP-W
**ORAC**, μM TE/g	DWE	3085 ± 55.53 ^a^	2544 ± 33.30 ^b^
	DWP	730.1 ± 10.41 ^a^	209.1 ± 6.75 ^b^
**ABTS^•^^⁺^**, μM TE/g	DWE	1667 ± 32.12 ^a^	1282 ± 19.33 ^b^
	DWP	394.7 ± 13.22 ^a^	105.4 ± 8.36 ^b^
**DPPH^•^**, μM TE/g	DWE	667.5 ± 20.74 ^a^	496.9 ± 18.58 ^b^
	DWP	158.0 ± 9.51 ^a^	40.85 ± 3.21 ^b^
**TPC**, mg GAE/g	DWE	154.2 ± 3.33 ^a^	131.6 ± 2.18 ^b^
	DWP	36.50 ± 1.91 ^a^	10.82 ± 0.95 ^b^
**Yields**, %		23.67 ± 0.11 ^a^	8.22 ± 0.13 ^b^

Values represented as mean ± standard deviation (*n* = 6); ^a, b^: the mean values in the rows for *Viburnum opulus* pomace ethanol (VOP-E) and water (VOP-W) extracts (dry weight of extract (DWE)) and initial material (dry weight of pomace (DWP)) followed by different letters are significantly different (*p* < 0.05).

**Table 2:** Identification and quantification of individual phenolic compounds of guelder rose berry pomace extracts.

Peak No.	Compound	Molecular Formula	*t*_R_ (min)	*m/z*, [M–H]^−^	MS/MS	VOP-E	VOP-W	VOP-E	VOP-W
						mg/100 g DWP		mg/100 g DWE	
***1***	Malic acid ^a,d,m,2^	C_4_H_6_O_5_	0.4	133.0144	71; 115	304.2 ± 0.25 ^a^	156.1 ± 0.003 ^b^	1285 ± 5.6 ^a^	1899 ± 10.88 ^b^
***2***	Citric acid ^a,d,s,2^	C_6_H_8_O_7_	0.4	191.0199	87; 111; 127; 173	-	9.95 ± 0.001	-	121.0 ± 0.45
***3***	Dihydroferulic acid 4-glucuronide ^b,c,d,l^	C_16_H_20_O_10_	0.6	371.0982	175	-	3.61 ± 0.003	-	43.92 ± 0.21
***4***	Procyanidin derivative ^b,c,d,p^	C_30_H_26_O_13_	0.7	593.1297	289; 305; 423; 467	-	1.36 ± 0.001	-	16.55 ± 0.10
***5***	Quinic acid ^a,d,q,2^	C_7_H_12_O_6_	0.8	191.0564	59; 85; 93; 127	0.24 ± 0.002 ^a^	2.39 ± 0.001 ^b^	1.01 ± 0.001 ^a^	29.08 ± 0.03 ^b^
**6**	Dihydroxybenzoic acid derivative ^b,c,d,m^	C_7_H_6_O_4_	0.9	153.0191	109	-	1.35 ± 0.001	-	16.42 ± 0.20
**7**	Iridoid derivative ^b,c,d,g^	C_16_H_26_O_14_	1.0	441.1248	113; 145; 173; 199; 217; 275; 395	2.53 ± 0.001	-	10.69 ± 0.01	-
***8***	Dihydroxybenzoic acid derivative ^b,c,d,m^	C_7_H_6_O_4_	1.0	153.0191	109	-	2.37 ± 0.01	-	28.83 ± 0.03
***9***	Malic acid dimethyl ester ^c,d,m^	C_6_H_10_O_5_	1.0	161.0453	133; 143	-	2.63 ± 0.01	-	31.99 ± 0.20
***10***	1-caffeoylquinic acid ^b,d,l^	C_16_H_18_O_9_	1.2	353.0875	133; 135; 161; 179; 191	-	2.95 ± 0.002	-	35.89 ± 0.02
***11***	NI ^g^	C_17_H_26_O_12_	1.2	421.1354	125; 135; 161; 179; 201; 215; 357; 375	1.04 ± 0.001 ^a^	0.02 ± 0.0001 ^b^	4.39 ± 0.01 ^a^	0.24 ± 0.01 ^b^
***12***	Hydroxybenzoic acid ^b,c,d,m^	C_7_H_6_O_3_	1.5	137.0243	93	-	2.38 ± 0.004	-	28.95 ± 0.03
***13***	Procyanidin dimer I (B2) ^a,b,d,p^	C_30_H_26_O_12_	1.6	577.1346	245; 289; 407; 425; 451	2.63 ± 0.003 ^a^	2.42 ± 0.0001 ^a^	11.11 ± 0.04 ^a^	29.44 ± 0.03 ^b^
***14***	Scopoletin-7-O-sophoroside ^b,d,p^	C_22_H_28_O_14_	1.6	515.1414	179; 191; 341; 353	1.96 ± 0.01	-	8.28 ± 0.01	-
***15***	(Epi)catechin-dihexoside ^b,d,r^	C_27_H_34_O_16_	1.7	613.1785	271; 289; 451; 503	1.06 ± 0.003 ^a^	3.09 ± 0.001 ^b^	4.48 ± 0.001 ^a^	37.59 ± 0.02 ^b^
***16***	Catechin ^a,b,d,k,2^	C_15_H_14_O_6_	2.0	289.0716	203; 205; 245	32.81 ± 0.02 ^a^	25.58 ± 0.54 ^b^	138.6 ± 0.01 ^a^	311.19 ± 0.55 ^b^
***17***	Gambiriin ^b,d,p^	C_30_H_28_O_12_	2.0	579.1515	205; 245; 289; 425	3.01 ± 0.01 ^a^	0.72 ± 0.003 ^b^	12.72 ± 0.01 ^a^	8.76 ± 0.02 ^b^
***18***	Chlorogenic acid ^a,b,d,l^	C_16_H_18_O_9_	2.2	353.0879	135; 161; 179; 191	214.8 ± 0.02 ^a^	99.20 ± 0.01 ^b^	907.5 ± 1.45 ^a^	1206 ± 4.89 ^b^
***19***	(Epi)catechin hexoside ^b,d,g^	C_21_H_24_O_11_	2.5	451.1245	245; 289; 341	1.74 ± 0.001 ^a^	1.29 ± 0.001 ^a^	7.35 ± 0.01 ^a^	15.69 ± 0.51 ^b^
***20***	Procyanidin dimer II ^b,d,p^	C_30_H_26_O_12_	2.5	577.1347	245; 289; 407; 425; 451	1.97 ± 0.004	-	8.32 ± 0.002	-
***21***	Cryptochlorogenic acid ^b,d,l^	C_16_H_18_O_9_	2.6	353.0877	135; 161; 173; 191	0.49 ± 0.003 ^a^	9.53 ± 0.43 ^b^	2.07 ± 0.001 ^a^	115.94 ± 0.23 ^b^
***22***	Procyanidin dimer III ^b,d,p^	C_30_H_26_O_12_	2.8	577.1348	245; 289; 407; 425; 451	8.89 ± 0.01 ^a^	8.23 ± 0.01 ^a^	37.56 ± 0.04 ^a^	100.1 ± 0.76 ^b^
***23***	NI ^p,1^	C_26_H_28_O_15_	3.4	579.1350	125; 161; 245; 289; 407; 409; 427; 453	-	2.54 ± 0.21	-	30.90 ± 0.01 ^b^
***24***	Epicatechin ^b,d,k,2^	C_15_H_14_O_6_	3.5	289.0714	203; 205; 245	8.52 ± 0.01 ^a^	16.08 ± 0.80 ^b^	67.93 ± 0.11 ^a^	195.6 ± 0.48 ^b^
***25***	Neochlorogenic acid ^b,d,l^	C_16_H_18_O_9_	3.5	353.0875	135; 161; 191	14.52 ± 0.02 ^a^	3.34 ± 0.002 ^b^	61.34 ± 0.12 ^a^	40.63 ± 0.57 ^b^
***26***	NI ^l^	C_17_H_26_O_9_	3.6	373.1503	-	3.37 ± 0.01	-	14.24 ± 0.01	-
***27***	Procyanidin C1 ^b,d,r^	C_45_H_38_O_18_	4.4	865.1984	287; 289; 407; 425; 451; 577; 695; 739	2.20 ± 0.003	-	9.29 ± 0.01	-
***28***	Cinchonain IIx ^b,d,r^	C_39_H_32_O_15_	4.6	739.1665	289; 325; 435; 451; 569; 587; 629	2.66 ± 0.002	-	11.24 ± 0.02	-
***29***	Dihydromyricetin ^b,d,l^	C_15_H_12_O_8_	5.3	319.0459	125; 153; 165; 193; 301	3.85 ± 0.004	-	16.27 ± 0.01	-
***30***	Quercetin pentoside hexoside I ^b,d,p^	C_26_H_28_O_16_	6.5	595.1304	151; 179; 301; 447	1.45 ± 0.21 ^a^	0.57 ± 0.003 ^b^	6.13 ± 0.01 ^a^	6.93 ± 0.02 ^a^
***31***	Cinchonain Ix ^b,d,g^	C_24_H_20_O_9_	7.0	451.1032	177; 189; 217; 231; 341	0.70 ± 0.001	-	2.96 ± 0.001	-
***32***	Rutin ^a,b,d,r^	C_27_H_30_O_16_	7.4	609.1453	151; 179; 301; 463	1.33 ± 0.002	-	5.62 ± 0.03	-
***33***	Quercetin-3-O-glucoside ^a,b,d,g^	C_21_H_20_O_12_	7.5	463.0879	151; 179; 301	1.85 ± 0.001	-	7.82 ± 0.02	-
***34***	Procyanidin dimer IV ^b,d,p^	C_30_H_26_O_12_	7.6	577.1348	245; 289; 407; 425; 451	0.48 ± 0.003	-	2.03 ± 0.001	-
***35***	Ethylchlorogenate ^b,d,l^	C_18_H_22_O_9_	8.0	381.1195	135; 161; 179	0.41 ± 0.006	-	1.73 ± 0.001	-
***36***	Cinchonain Ix ^b,d,g^	C_24_H_20_O_9_	8.3	451.1033	177; 189; 217; 231; 341	59.84 ± 1.41 ^a^	15.53 ± 0.66 ^b^	252.8 ± 0.63 ^a^	188.9 ± 1.79 ^b^
***37***	Cinchonain Ix ^b,d,g^	C_24_H_20_O_9_	8.7	451.1034	177; 189; 217; 231; 341	3.42 ± 0.01 ^a^	0.10 ± 0.0001 ^b^	14.45 ± 0.88 ^a^	1.22 ± 0.001 ^b^
***38***	Quercetin pentoside hexoside II ^b,d,p^	C_28_H_36_O_14_	8.9	595.2670	151; 179; 301	0.77 ± 0.001	-	3.25 ± 0.01	-
***39***	Secologanate ^b,d,l^	C_16_H_22_O_10_	8.9	373.1141	123; 149; 167; 193; 211	2.77 ± 0.04	-	11.70 ± 0.01	-
***40***	(Epi)catechin dimer monoglycoside ^b,d,r^	C_31_H_48_O_20_	9.0	739.2665	177; 289; 339; 451; 577	1.28 ± 0.01	-	5.41 ± 0.56	-
***41***	Cinchonain Ix ^b,d,g^	C_24_H_20_O_9_	9.1	451.1038	177; 189; 217; 231; 341	1.98 ± 0.001 ^a^	3.45 ± 0.001 ^b^	8.37 ± 0.04 ^a^	41.97 ± 1.34 ^b^
***42***	Viburtinoside derivative ^b,d,e,r^	C_26_H_40_O_16_	9.2	607.2240	231; 339; 441; 501; 561	3.56 ± 0.01	-	15.04 ± 0.03	-
***43***	Viburtinoside derivative ^b,d,e,r^	C_26_H_40_O_16_	9.3	607.2242	231; 339; 441; 501; 561	1.21 ± 0.01	-	5.11 ± 0.01	-
***44***	Opulus iridoid III ^b,d,e,r^	C_33_H_50_O_21_	10.0	781.2775	453; 513; 573; 615; 633; 675; 735	4.11 ± 0.001	-	17.36 ± 0.12	-
***45***	Opulus iridoid II ^b,d,e,r^	C_28_H_42_O_17_	10.2	649.2346	381; 483; 501; 543; 603	10.51 ± 0.53	-	44.40 ± 0.99	-

Superscript letters with the compound name: ^a^ confirmed by a standard; ^b^ confirmed by a reference; ^c^ confirmed by parent ion mass using a free chemical database (Chemspider, PubChem, Metlin); ^d^ confirmed by MS/MS fragmentation; ^e^ detected as formic acid adduct; ^m, s, q, p, k, l, r^, and ^g^ based on the calibration curve obtained by using malic (LOD 2.30; LOQ 6.98), citric (LOD 4.57; LOQ 13.84), quinic (LOD 1.48; LOQ 4.47), proanthocyanidin B2 (LOD 16.24; LOQ 49.20), catechin (LOD 15.33; LOQ 46.46), chlorogenic acid (LOD 14.97; LOQ 45.38), rutin (LOD 0.58; LOQ 1.77), and quercetin-3-O-glucoside (LOD 0.81; LOQ 2.45), respectively; ^1^ collision energy 20 V, ^2^ collision energy 25 V; ^a, b^ the mean values in the rows for *V. opulus* pomace ethanol (VOP-E) and water (VOP-W) extracts (DWE) followed by different letters are significantly different (*p* < 0.05); NI-: not identified.

**Table 3:** Structures of some bioactive compounds identified in guelder rose berry pomace extracts.

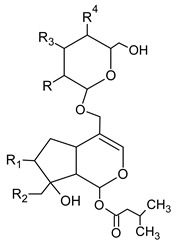	Viburtinoside derivatives								
		R	R_1_	R_2_			R_3_		R_4_
	IV	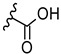	OH	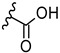			OH		OH
	V	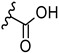	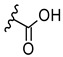	OH			OH		OH
	Opulus iridoids								
	II	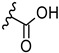	OH	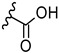			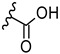		OH
	III	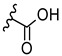	OH	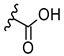			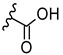		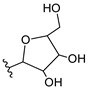
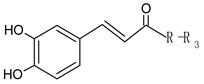		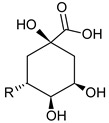	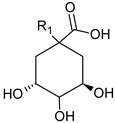			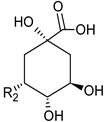		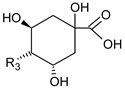	
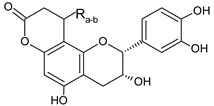 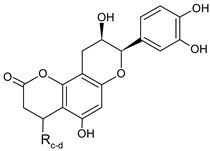		Chlorogenic acid	1-*O*-caffeoylquinic acid			Cryptochlorogenic acid		Neochlorogenic acid	
		Cinchonain I							
		a	b		c			d	
		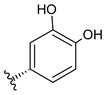	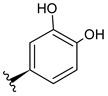		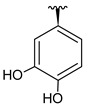			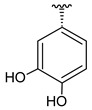	
		Cinchonain II							
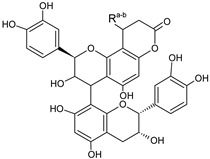		a				b			
		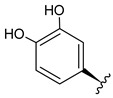				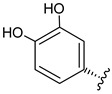			
Quertecin-3-*O*-glucoside	Secologanate		Gambiriin				Scopoletin-7-*O*-sophoroside		
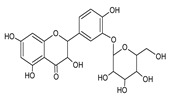	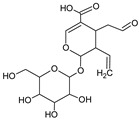		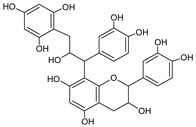				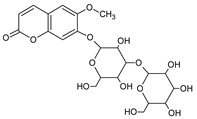		

## Supplementary Materials

The following are available online at https://www.mdpi.com/2304-8158/9/10/1413/s1.
