# Supplementary material for: Valorization of European Cranberry Bush (Viburnum opulus L.) Berry Pomace Extracts Isolated with Pressurized Ethanol and Water by Assessing Their Phytochemical Composition, Antioxidant, and Antiproliferative Activities
Source: Foods. 2020 Oct 6;9(10):1413. doi: 10.3390/foods9101413 (PMC7601694; doi:10.3390/foods9101413)

*Supplementary material*

# Valorisation of European cranberry bush (*Viburnum opulus* L.) berry pomace by recovery and evaluation of valuable ingredients for functional foods and nutraceuticals

**Lijana Dienaitė [1], Milda Pukalskienė [1], Carolina V. Pereira [2], Ana A. Matias [2], Petras Rimantas Venskutonis [1,*]**

[1] Department of Food Science and Technology, Kaunas University of Technology, Radvilėnų pl. 19, Kaunas LT-50254, Lithuania
[2] IBET,—Instituto de Biologia Experimental e Tecnológica, Food & Health Division Apartado 12, 2780-901 Oeiras, Portugal
* Correspondence: rimas.venskutonis@ktu.lt; tel.: +370 699 40978, +370 37 456647.

Received: date; Accepted: date; Published: date

## Materials and Methods

### *Chemicals and cells*

Folin-Ciocalteu reagent, Trolox (6-hydroxyl-2,5,7,8-*tetra*-methylchroman-2-carboxylic acid), DPPH• (2,2-diphenyl-1-picrylhydrazyl radical, 98%), gallic acid, $KH_2PO_4$ (monopotassium phosphate), KCl (potassium chloride), NaCl (sodium chloride), formic acid (98%), ABTS, (2,2'-azino-*bis*-3-ethylbenzothiazoline-6-sulfonic acid, 98%), $K_2S_2O_8$ (potassium persulfate), ammonium hydroxide, AAPH (2',2'-azo-*bis*-(2-amidinopropane) dihydrochloride), HPLC grade and LS-MS grade acetonitrile, HPLC grade formic acid (98%) were obtained from Sigma-Aldrich (Darmstadt, Germany). Disodium fluorescein, $Na_2HPO_4{\cdot}2H_2O$ (sodium phosphate dibasic dihydrate) and ethanol (99.9%) were from TCI Europe (Antwerp, Belgium), Riedel-de-Haen (Seelze, Germany) and Scharlau (Barcelona, Spain), respectively. $Na_2CO_3$, 2′,7′-dichlorofluorescin diacetate (DCFH-DA), quercetin (95%) were from Sigma-Aldrich (St. Quentin Fallavier, France). Ultra-pure water was produced in a Simplicity 185 system (Millipore, Billerica, MA, USA). Analytical grade methanol was from StanLab (Lublin, Poland). The standards used for UPLC analysis, namely malic acid, fructose, glucose, sucrose, rutin, quinic, citric and chlorogenic acids were from Supelco Analytical (Bellefonte, PA, USA); catechin, proanthocyanidin B2 and quercetin-3-O-glucoside from Extrasynthese (Genay Cedex, France).

Human Caco-2 (epithelial colorectal adenocarcinoma) and HT29 (human colon cancer) cell lines were purchased from DSMZ (Braunschweig, Germany) and ATCC (Manassas, VA, USA), respectively. The cell culture medium and supplements were purchased from Invitrogen (Gibco, Paisley, UK). Phosphate buffered saline (PBS) was obtained from Sigma-Aldrich (St. Louis, MO, USA) and cell viability was assessed using a CellTiter 96® AQueous One Solution Cell Proliferation Assay (Promega, Madison, WI, USA).

### *Plant material, extraction procedure and analysis methods*

Dry VOP was obtained from BestBerry (Auce, Latvia) and ground in a laboratory mill Vitek (An-Der, Austria) by using 0.5 mm size sieve. VOP powder was extracted by SFE-$CO_2$ in a 100 mL extractor (Applied Separations, Allentown, PA, USA) to remove lipophilic fraction. For PLE, 10 g of defatted pomace powder were mixed with 4 g of diatomaceous earth, placed in 66 mL extraction cells and firstly extracted with ethanol then consecutively extracted with water in an accelerated solvent extraction apparatus ASE350 (Dionex, Sunnyvale, CA, USA) at constant 10.3 MPa pressure and temperature (70 °C for ethanol and 120 °C for water) using 15 min static and a 90 s purge time for each extraction cycle (total 3 cycles). Ethanol was evaporated at 40 °C in a Rotavapor R-114 (Büchi, Flawil, Switzerland), while residual water was removed by

freeze-drying in a Maxi Dry Lyo (Hetto-Holton AIS, Allerod, Denmark). The extracts were weighed and stored at -18 °C in a freezer until further analysis. Further they are abbreviated as VOP-E and VOP-W.

*Proximate analysis of VOP*

Chemical composition of VOP was determined according to the procedures established by the Association of Official Analytical Chemists: moisture by drying at 105 °C to constant weight; ash by mineralizing in a muffle furnace F-A1730 (Thermolyne Corp., Dubuque, IA, USA) at 500 °C for 3 h; proteins by Kjeldahl method in a nitrogen analyzer (Leco Instruments Ltd., Mississauga, ON, Canada) using a conversion factor of 6.25; crude lipids by Soxhlet extraction with hexane for 6 h. The rest of dry matter was assigned to carbohydrates. Each determination was carried out in triplicate.

*Total phenolic content (TPC) and antioxidant capacity*

Folin–Ciocalteau (TPC), DPPH, ABTS and ORAC assays were used for evaluating antioxidant potential of VOP extracts. TPC was performed as described by Singleton and Rossi with some modifications. 30 μL of extract solutions were mixed with 150 μL Folin-Ciocalteu reagent (1:10 in distilled water) with 120 μL 7% $Na_2CO_3$ solution in a 96-well microplate and the absorbance was measured at 765 nm after 30 min in a FLUOstar Omega Reader (BMG Labtech, Offenburg, Germany). Gallic acid solutions (10–250 μg/mL) were used for the calibration curve. TPC was expressed in mg of gallic acid equivalents in g of extract and pomace, GAE/g DWE (dry weight of extract) and DWP (dry weight of pomace), respectively.

Re et al. method with some modifications was used for $ABTS^{•+}$ decolourisation assay. 6 μL of sample were added to 294 μL of $ABTS^{•+}$ working solution. The $ABTS^{•+}$ solution was prepared by mixing fifty mL of ABTS (2 mM) with 200 μL of potassium persulfate (70 mM); the mixture was kept in the dark for 15–16 h before use. The working solution was prepared by diluting with PBS (prepared from 8.18 g of NaCl, 0.27 g of KH2PO4, 1.78 g of Na2HPO4 × 2 H2O and 0.15 g of KCl in 1 L of distilled water) to obtain the absorbance of 0.800 ± 0.030 at 734 nm. The absorbance was measured in a 96-well microplate using a FLUOstar Omega Reader during 30 min at 734 nm A series of Trolox solutions (399–1198 μM/L;) were used for calibration. The results were expressed as μM TE/g DWE and DWP.

Brand-Williams et al. method was applied for $DPPH^{•}$- scavenging assay with some modifications. 8 μL of sample were mixed with 292 μL of $DPPH^{•}$ methanolic solution (0.0059 g/250 mL). The working solution was prepared by diluting with methanol to obtain the absorbance of 0.7 at 515 nm. After mixing, the microplate was placed in a reader, shaken for 30 s, incubated for 60 min and the absorbance read at 515 nm. The results were calculated as in the ABTS assay.

ORAC assay was performed by using fluorescein as a fluorescent probe and AAPH as a peroxyl radical generator. 25 μL of sample were pipetted into 150 μL (14 μM) fluorescein solution and after incubation during 15 min at 37 °C 25 μL of AAPH (240 mM) were added. The fluorescence was recorded every cycle (in total, 120 cycles) using 485 excitation and 530 emission fluorescence filters. Antioxidant curves (fluorescence versus time) were first normalized and from the normalized curves the net area under the fluorescein decay curve (AUC) was calculated by the formula: AUC = (1 + f1 f0 + f2 f0… fi f0 ) × CT (1) where f0 is the initial fluorescence reading at 0 min, fi is the fluorescence reading at time i and CT is cycle time in minutes. The final ORAC values were calculated by using regression equation between Trolox concentration and the net AUC. Trolox solutions (0–250 μM) were used for the calibration. The results were expressed as μM TE/g DWE and DWP. Each measurement was carried out in six replicates.

*HPLC-DPPH•-scavenging on-line*

HPLC-$DPPH^{•}$-scavenging online analysis was carried out on a Waters HPLC system (Waters Corporation, Milford, MA, USA) with minor changes. The mobile phase consisted of 0.4% formic acid in water (A) and acetonitrile (B). The elution started with 90% A, then changed to 60% in 45 min, after that in 50 min A decreased to 5%, then A increased to 90% in 53 min, was hold for 3 min and finally it was returned to initial conditions in 2 min and the column was equilibrated for 5 min. Decrease of absorbance after the reaction of radical scavengers with $DPPH^{•}$ was measured at 515 nm with variable-wavelength Shimadzu

SPD–20A UV detector (Shimadzu Corporation, Kyoto, Japan), while identification of compounds was performed by using Waters Acquity UPLC system (Milford, MA, USA).

*Cell Culture and Sample Preparation*

Water and ethanol VOP extracts were solubilized in DMSO (200 mg/mL) and ethanol (100 mg/mL), respectively and stored at –20 °C protected from light. Cell-based assays were performed using maximum concentrations of solvents, 1% and 5% for DMSO and ethanol, respectively. Caco-2 cell lines were cultured in RPMI-1640 medium supplemented with 10% of heat-inactivated foetal bovine serum (FBS) and 1% penicillin-streptomycin (PS). The cells were maintained at 37 °C with 5% $CO_2$ in a humidified incubator and routinely grown as a monolayer in 75 mL culture flasks.

*Cellular Antioxidant Activity (CAA) Assay*

In Caco-2 monolayers 50 μL of PBS, sample and standard (quercetin, 2.5-20 μM) solution and 50 μL of DCFH-DA solution (50 μM) were added and incubated for 60 min at 37 °C, 5% $CO_2$. Afterwards, 100 μL of AAPH (12 mM) solution was added to each well containing PBS/quercetin standards/samples, while 100 μL of PBS were added to the blank wells. Fluorescence kinetics was recorded every 5 min during 60 min at excitation and emission wavelengths of 485 nm and 540 nm, respectively in a Microplate Fluorimeter FLx800 (Bioteck Instruments, VT, USA). CAA values were expressed as μM of QE/g of extract of independent experiments performed in triplicates.

*Antiproliferative activity assay*

Antiproliferative effect of VOP and standard compounds was evaluated in HT29 cells. Briefly, the cells were seeded at a density of $1\times10^4$ cells/well in a 96-well culture plate. After 24 hours the cells were incubated with different concentrations of the samples diluted in a culture medium. Cell proliferation was measured after 24 hours using MTS reagent, as mentioned above. The results were expressed in terms of percentage of living cells relative to the control. Three independent experiments were performed in triplicate.

**$MS^1$ spectra of the compounds listed in Table 2**

(MS spectra have been taken from the PC monitor by camera)

Malic (**1**) and Citric (**2**) acids

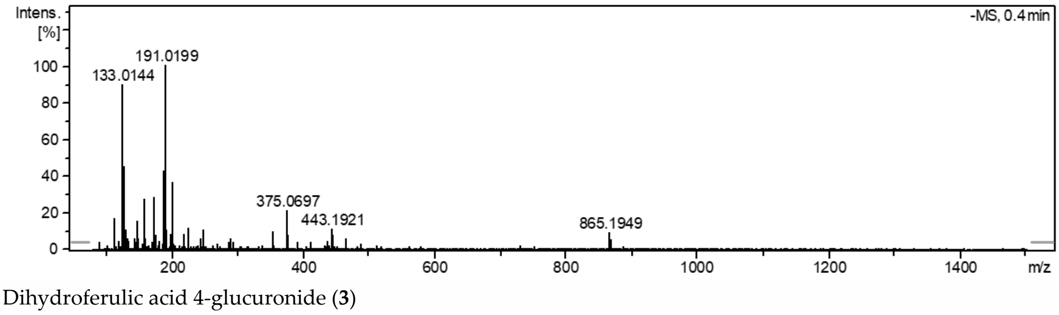


Dihydroferulic acid 4-glucuronide (**3**)

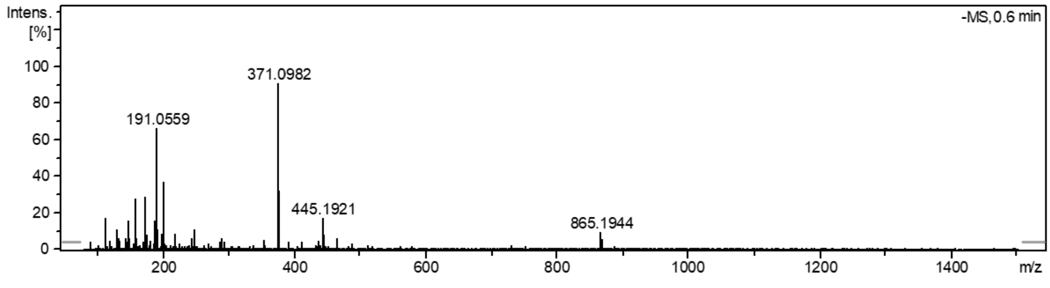

Procyanidin derivative (**4**)

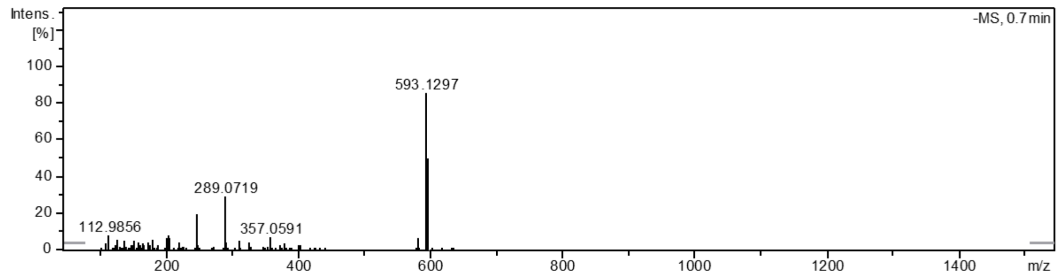


Quinic acid (**5**)

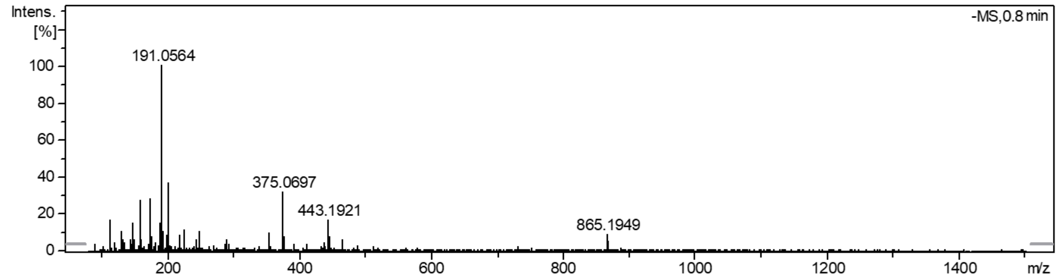


Dihydroxybenzoic acid derivative (**6**)

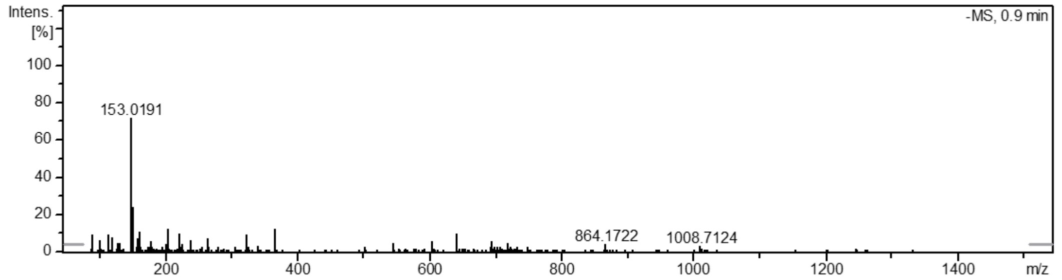


Iridoid derivative (**7**)

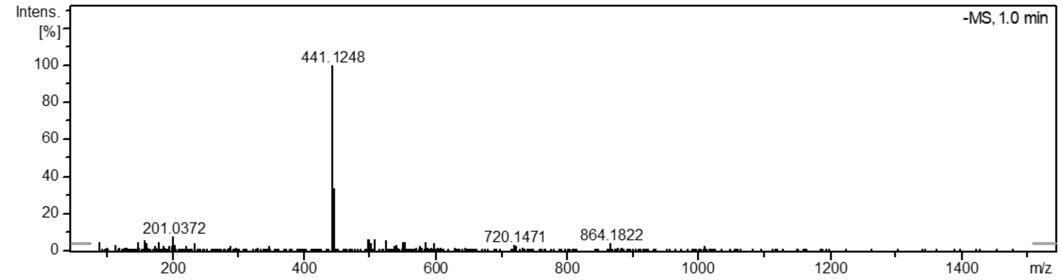


Dihydroxybenzoic acid derivative (**8**) and Malic acid dimethyl ester (**9**)

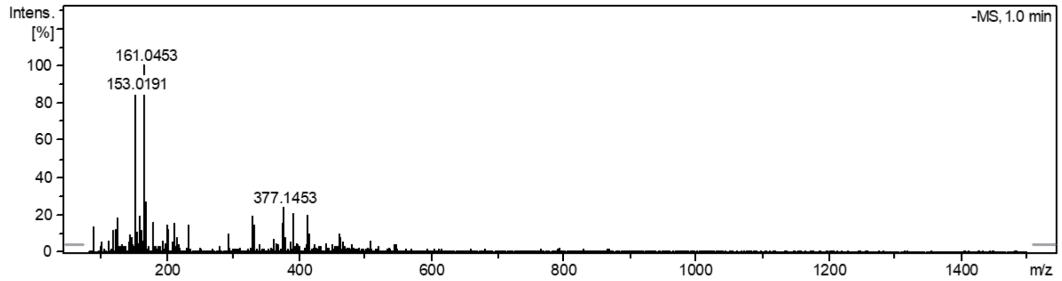


1-caffeoylquinic acid (**10**)

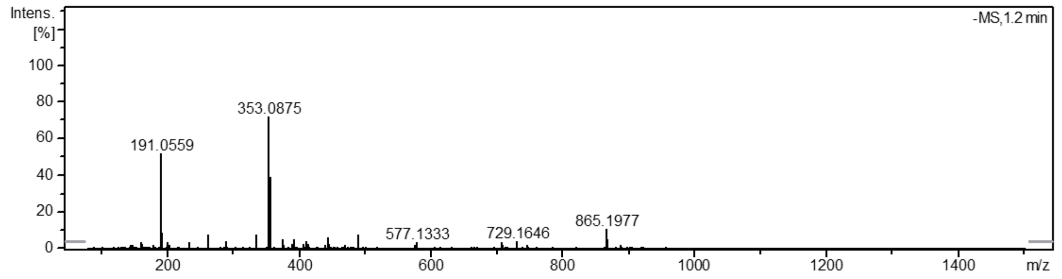

NI (**11**)

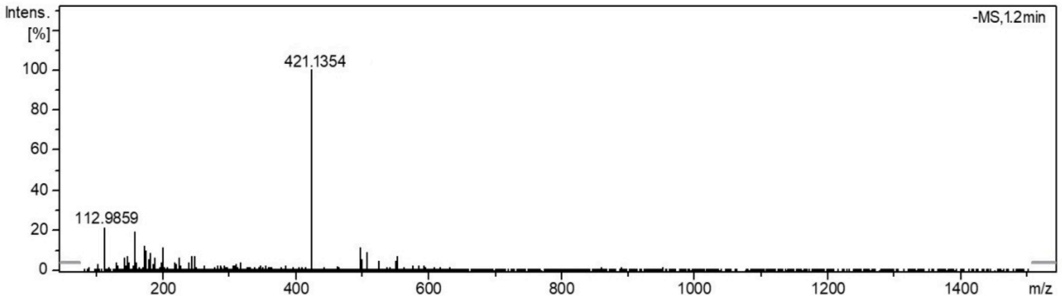


Hydroxybenzoic acid (**12**)

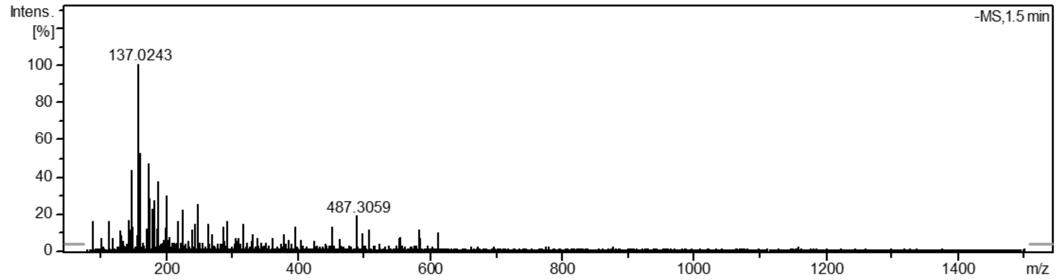


Procyanidin dimer I (B2) (**13**) and Scopoletin-7-O-sophoroside (**14**)

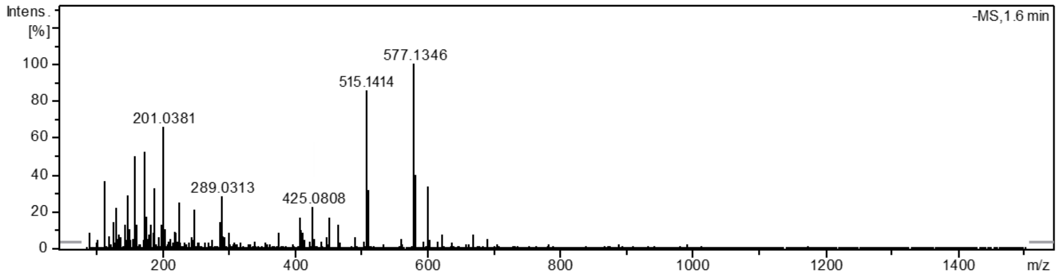


(Epi)catechin-dihexoside (**15**)

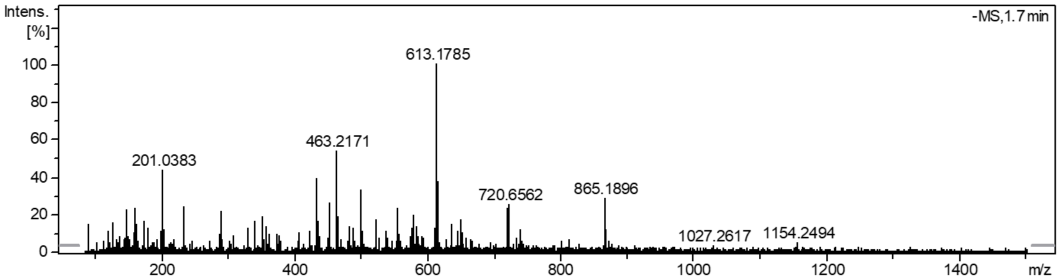


Catechin (**16**) and Gambiriin (**17**)

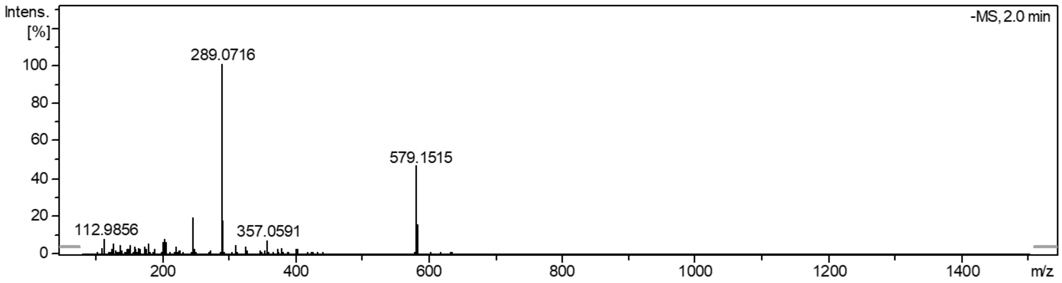

Chlorogenic acid (**18**)

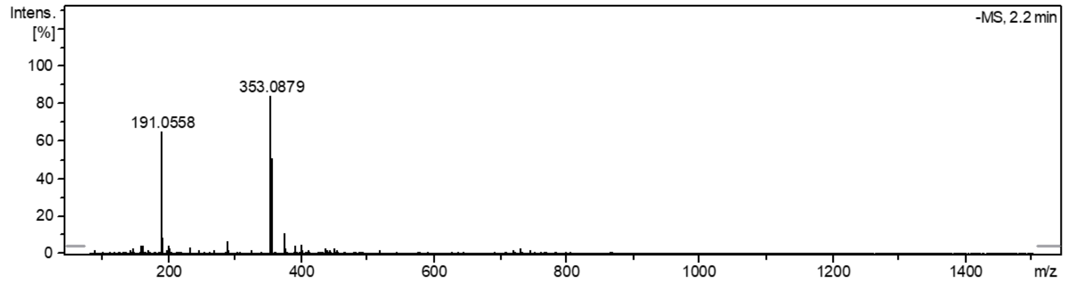


(Epi)catechin hexoside (**19**) and Procyanidin dimer II (**20**)

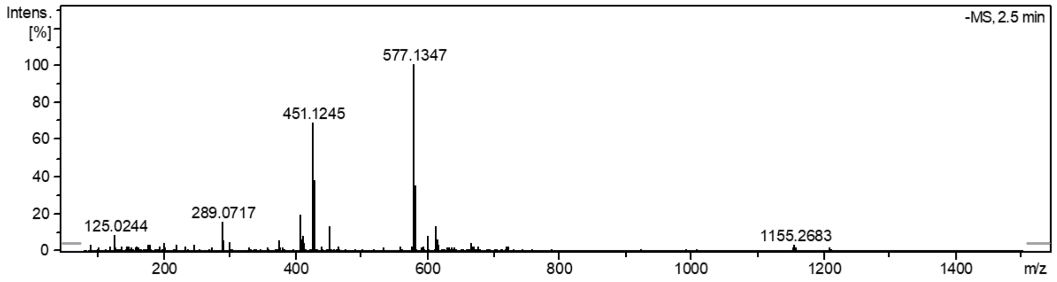


Cryptochlorogenic acid (**21**)

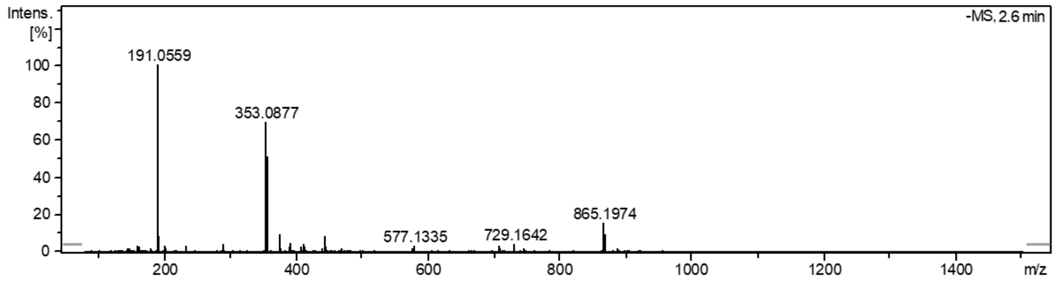


Procyanidin dimer III (**22**)

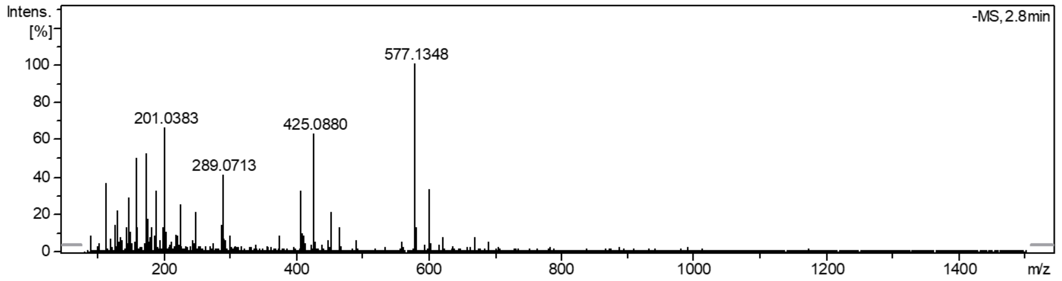


NI (**23**)

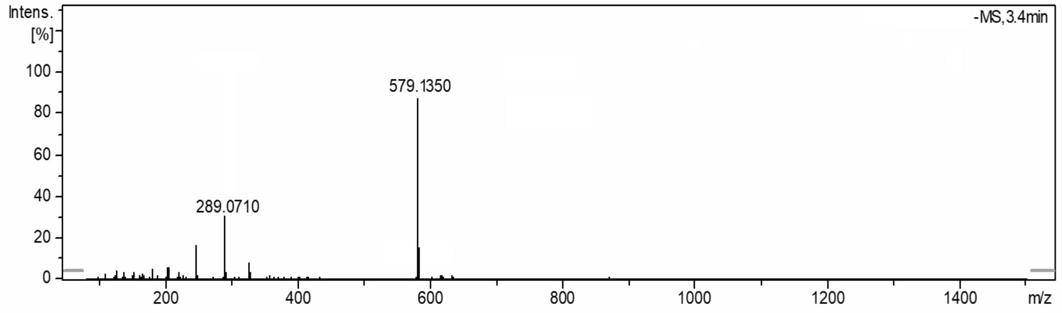

Epicatechin (**24**) and Neochlorogenic acid (**25**)

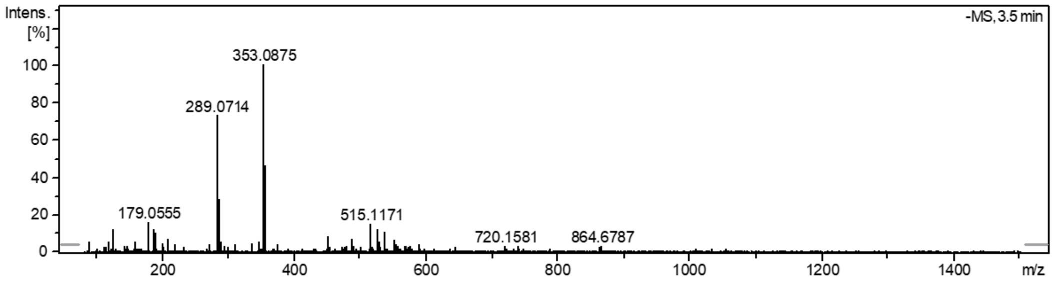


NI (**26**)

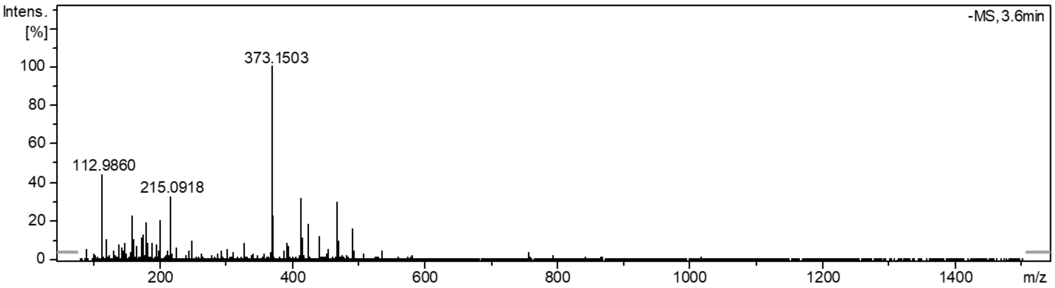


Procyanidin C1 (**27**)

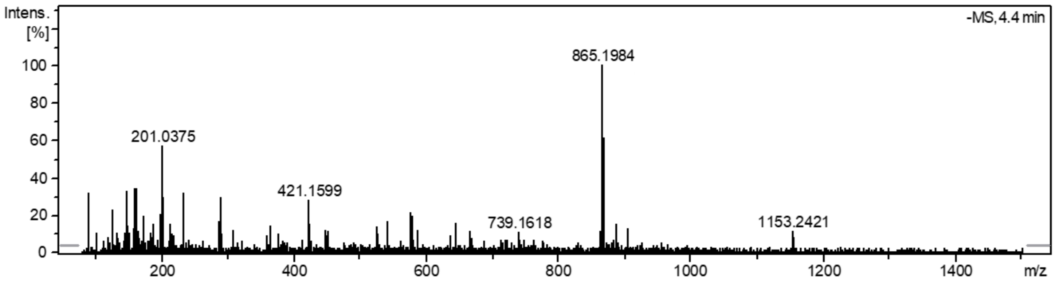


Cinchonain IIx (**28**)

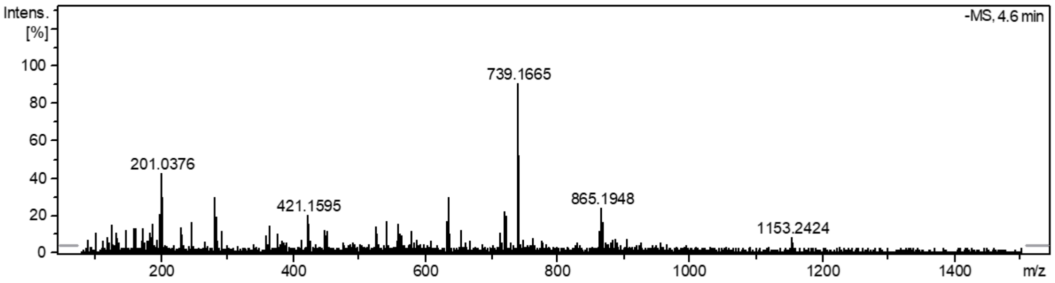


Dihydromyricetin (**29**)

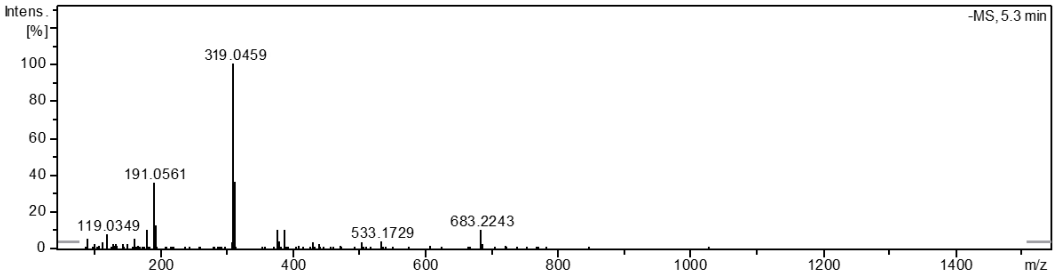

Quercetin pentoside hexoside I (**30**)

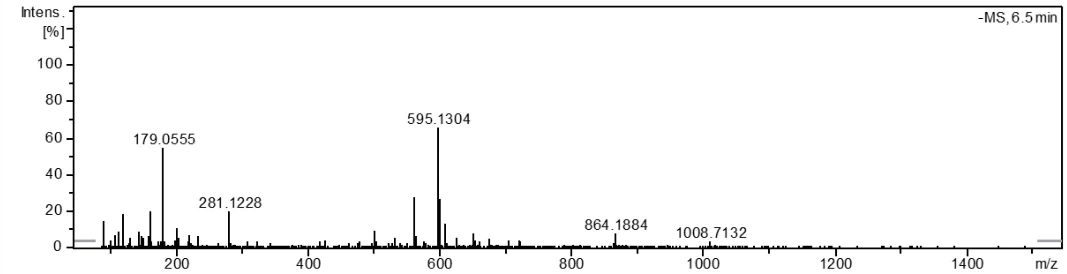


Cinchonain Ix (**31**)

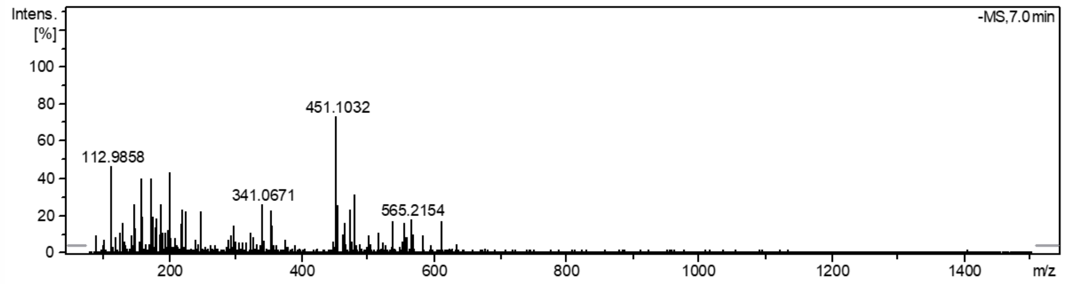


Rutin (**32**)

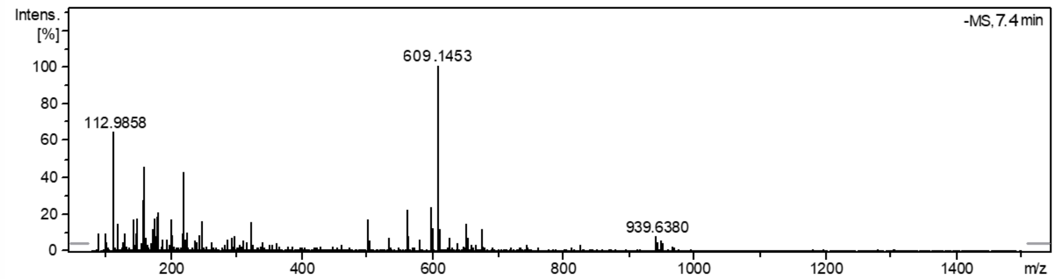


Quercetin-3-O-glucoside (**33**)

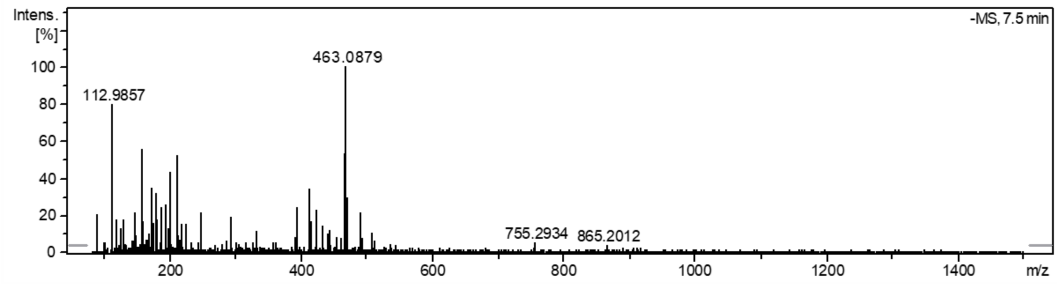


Procyanidin dimer IV (**34**)

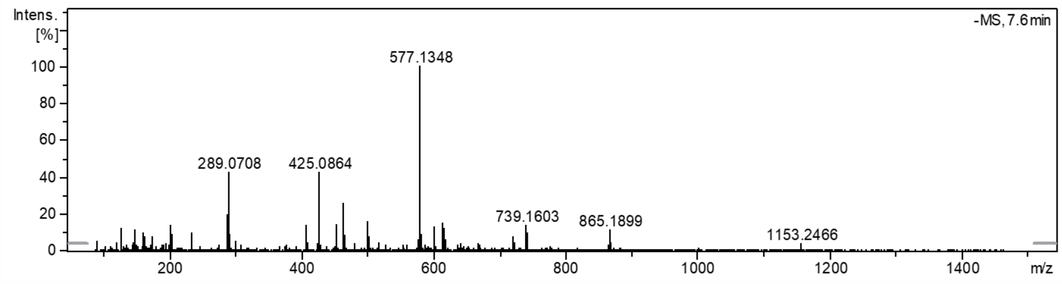


Ethylchlorogenate (**35**)

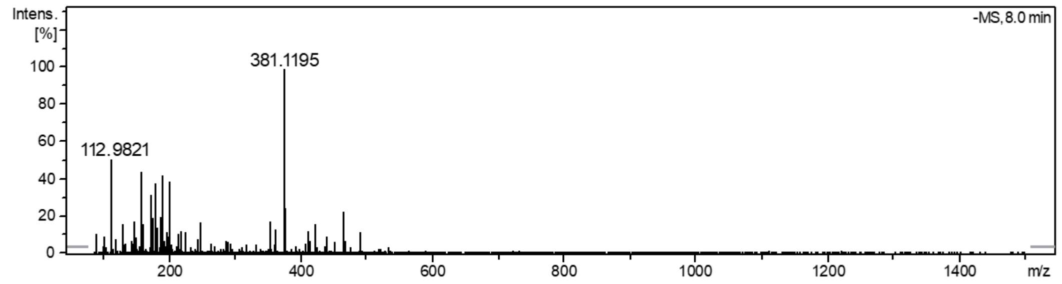

Cinchonain Ix (**36**)

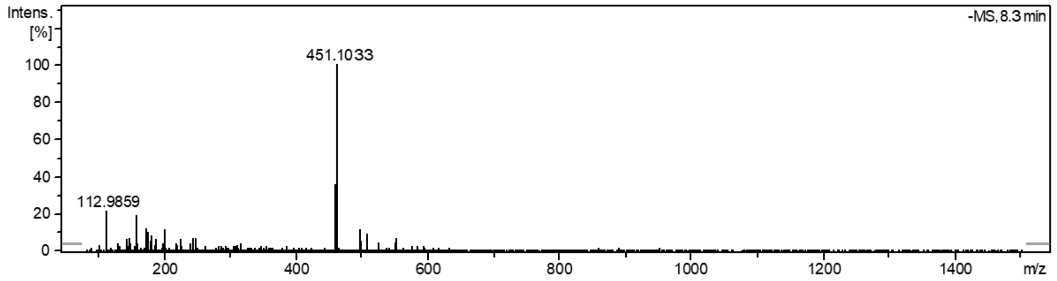


Cinchonain Ix (**37**)

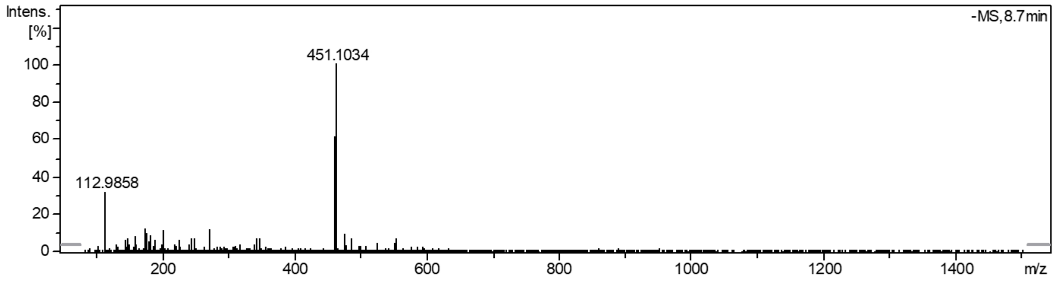


Quercetin pentoside hexoside II (**38**) and Secologanate (**39**)

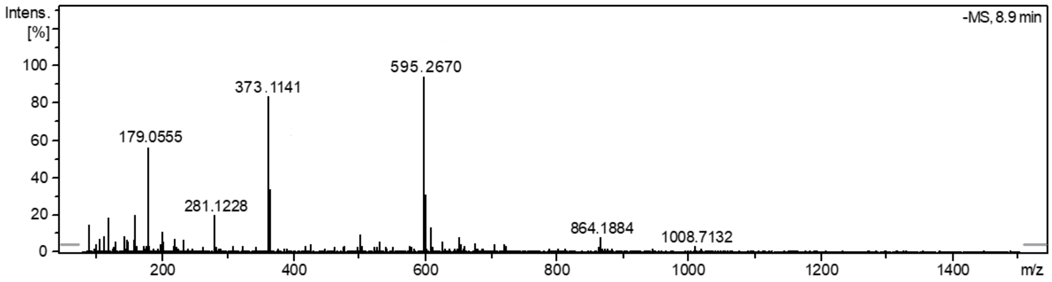


(Epi)catechin dimer monoglycoside (**40**)

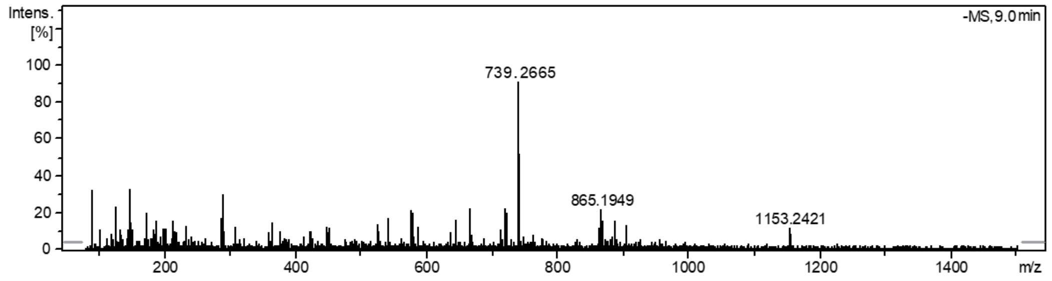


Cinchonain Ix (**41**)

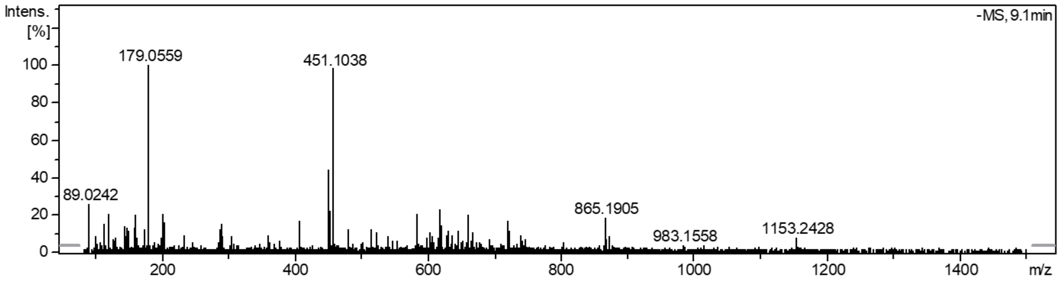


Viburtinoside derivative (**42**)

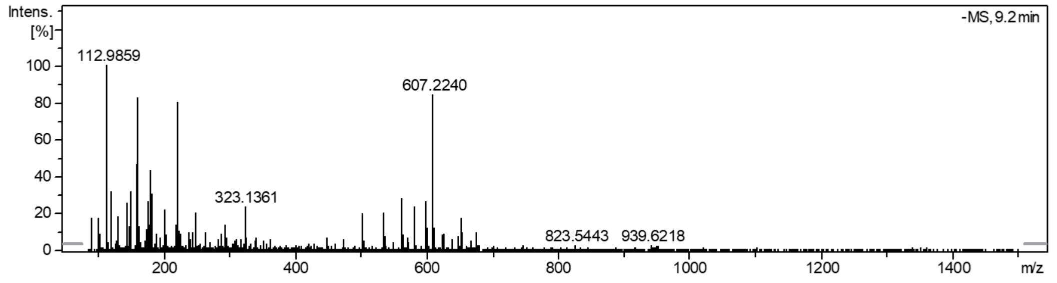

Viburtinoside derivative (**43**)

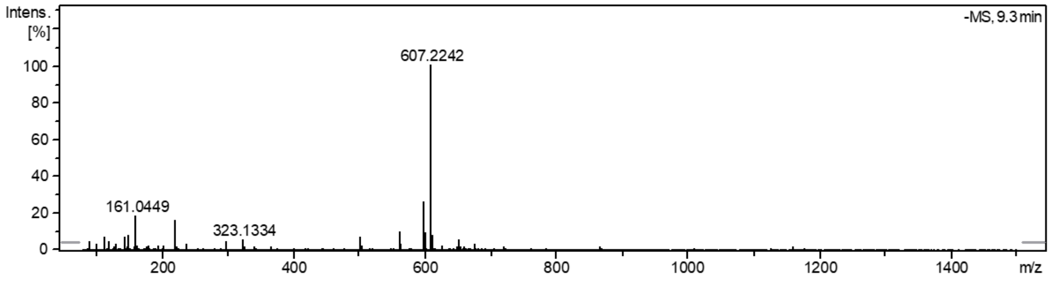


Opulus iridoid III (**44**)

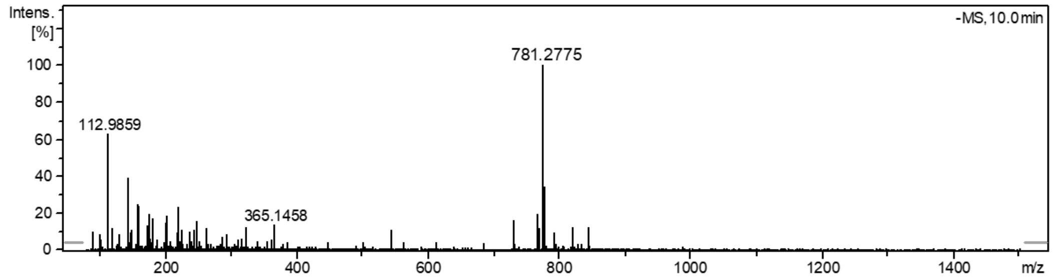


Opulus iridoid II (**45**)

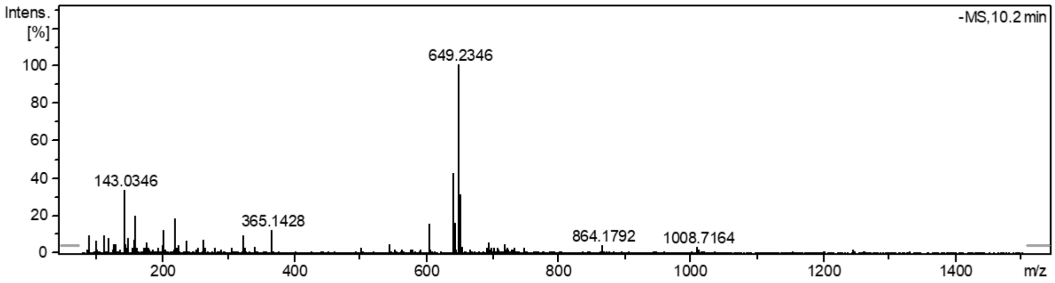

**MS² spectra of the compounds listed in Table 2**

Spectra of: Malic acid (**1**)

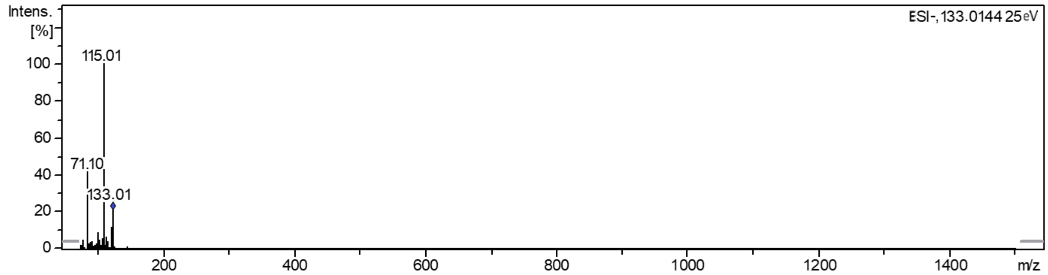


Citric acid (**2**)

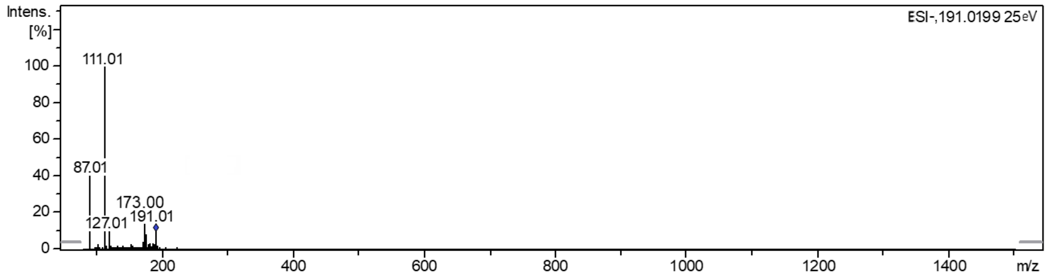


Dihydroferulic acid 4-glucuronide (**3**)

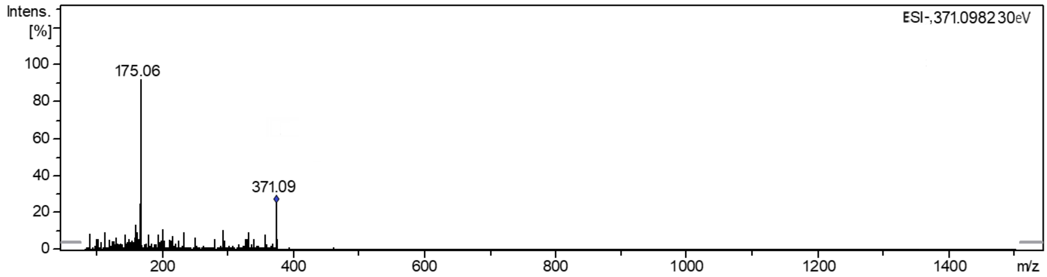


Procyanidin derivative (**4**)

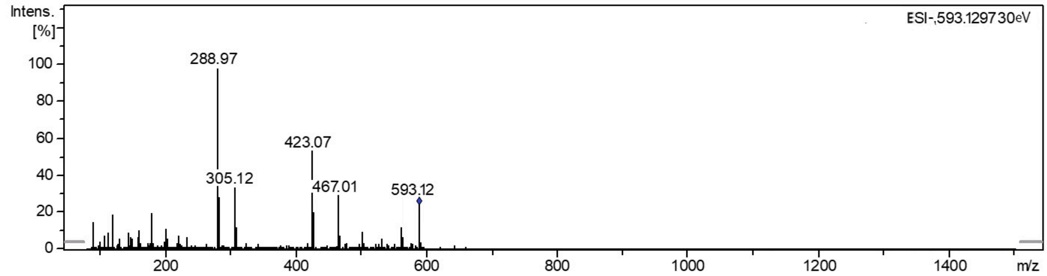


Quinic acid (**5**)

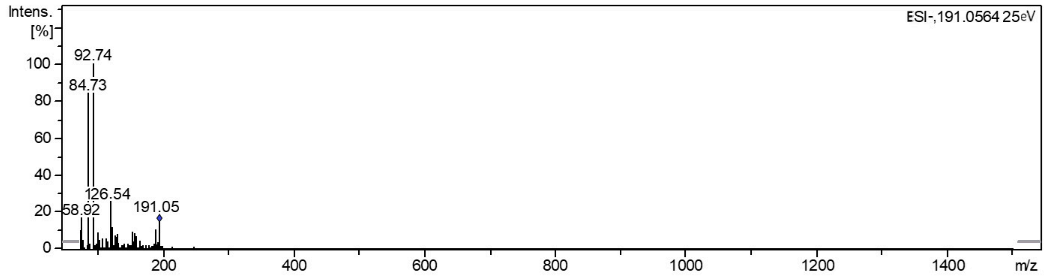

Dihydroxybenzoic acid derivative (**6**)

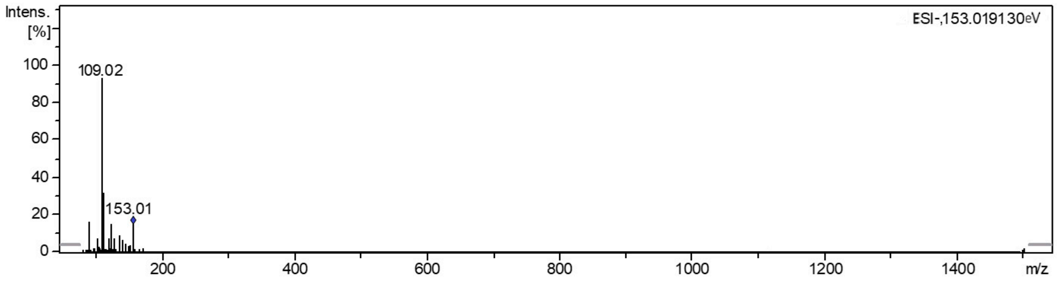


Iridoid derivative (**7**)

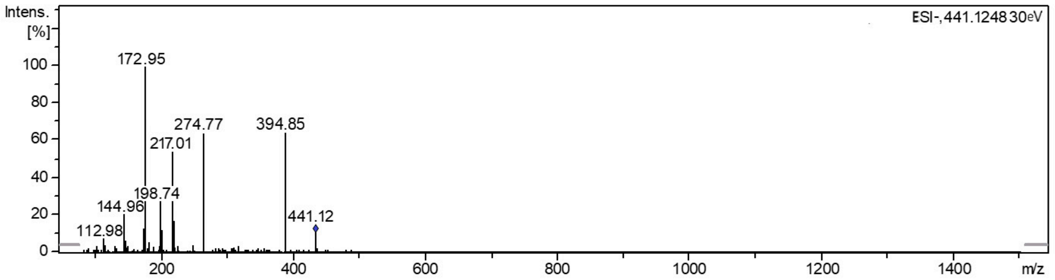


Dihydroxybenzoic acid derivative (**8**)

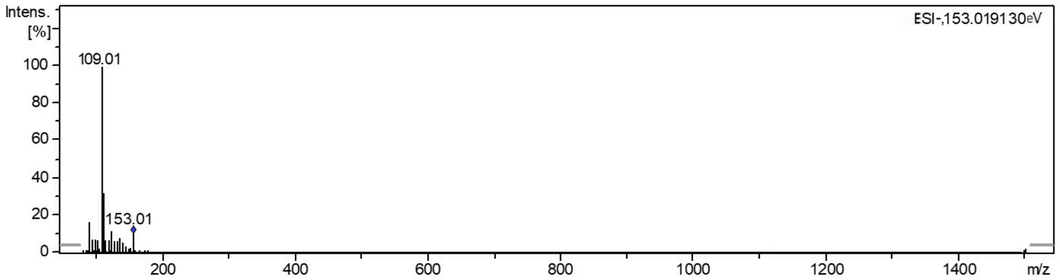


Malic acid dimethyl ester (**9**)

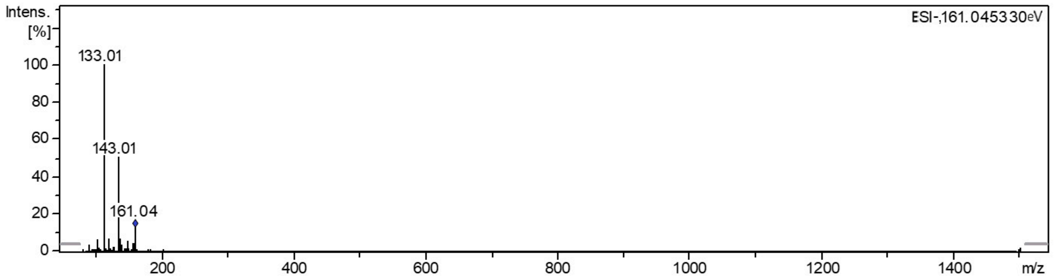


1-caffeoylquinic acid (**10**)

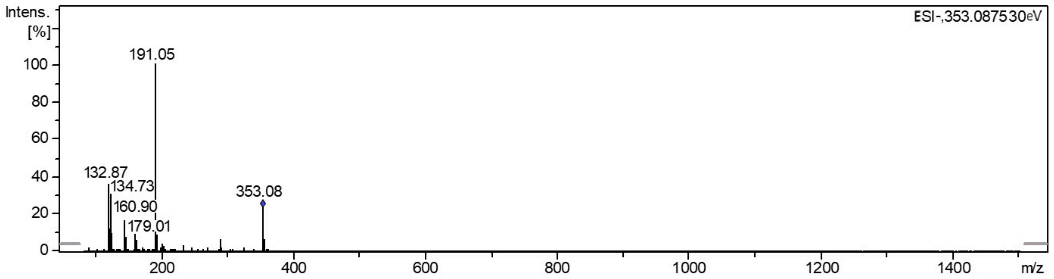

NI (**11**)

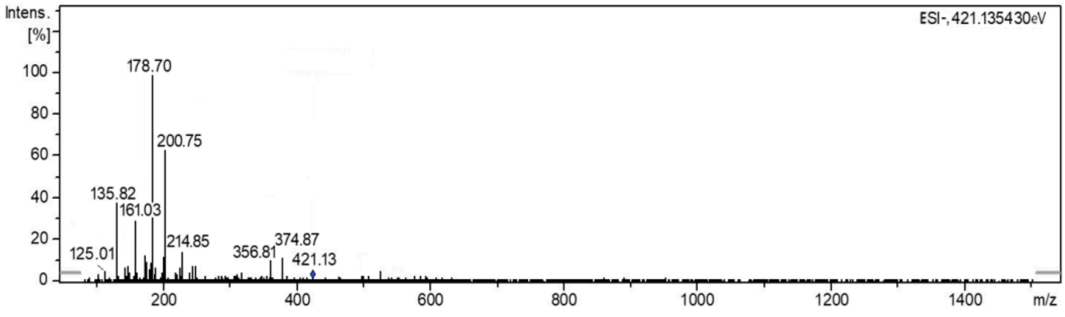


Hydroxybenzoic acid (**12**)

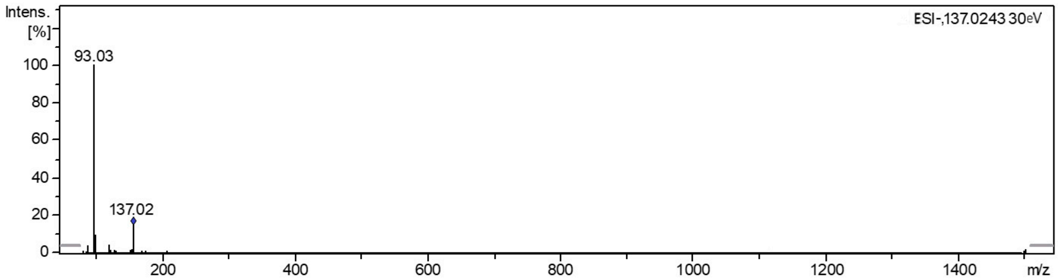


Procyanidin dimer I (B2) (**13**)

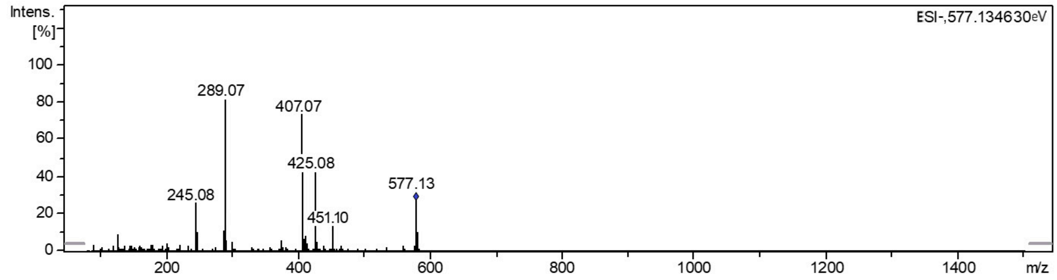


Scopoletin-7-O-sophoroside (**14**)

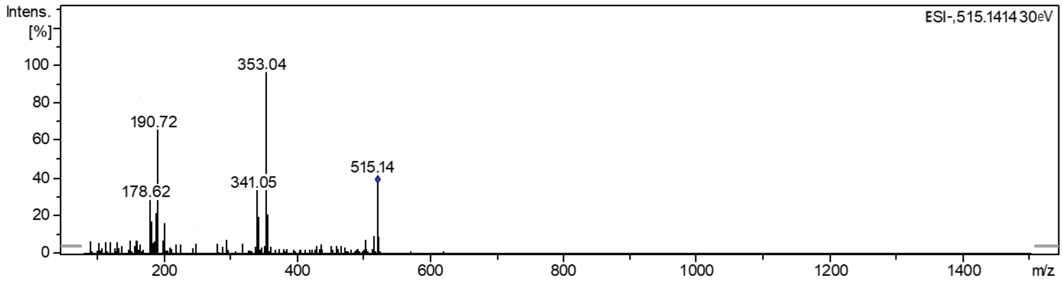


(Epi)catechin-dihexoside (**15**)

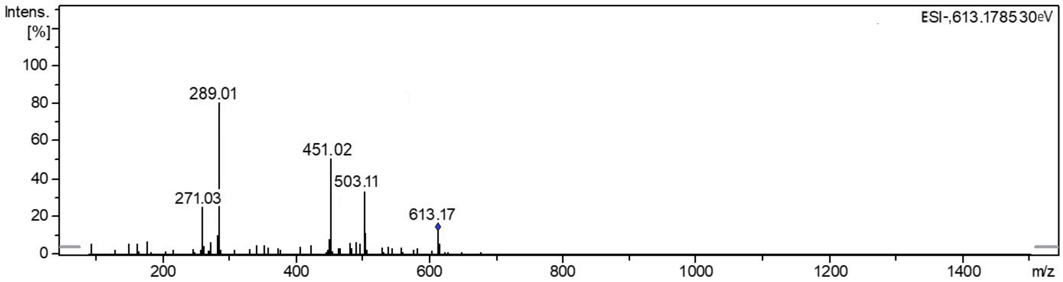

Catechin (**16**)

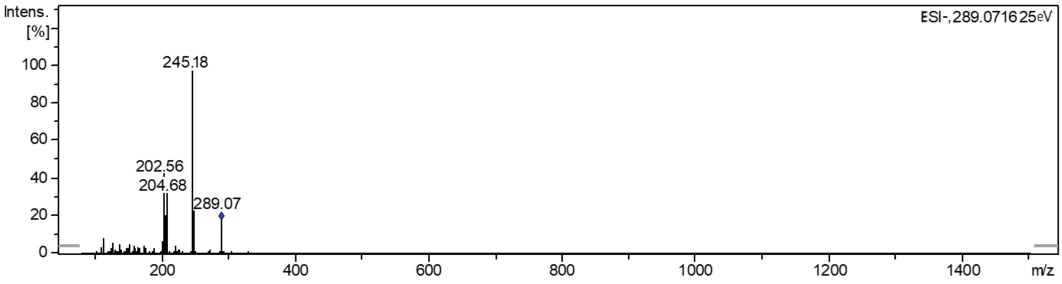


Gambiriin (**17**)

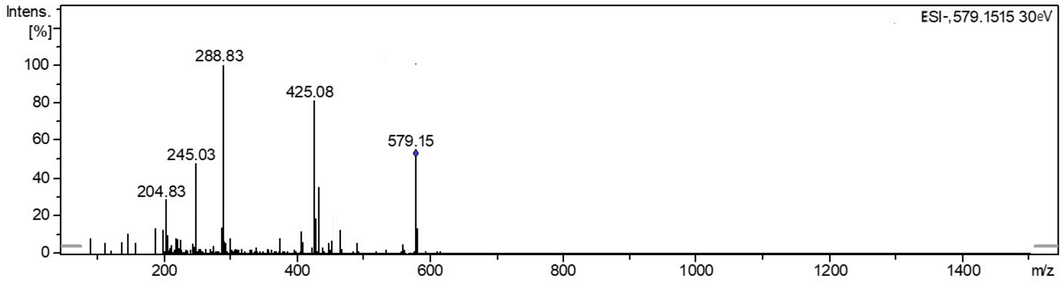


Chlorogenic acid (**18**)

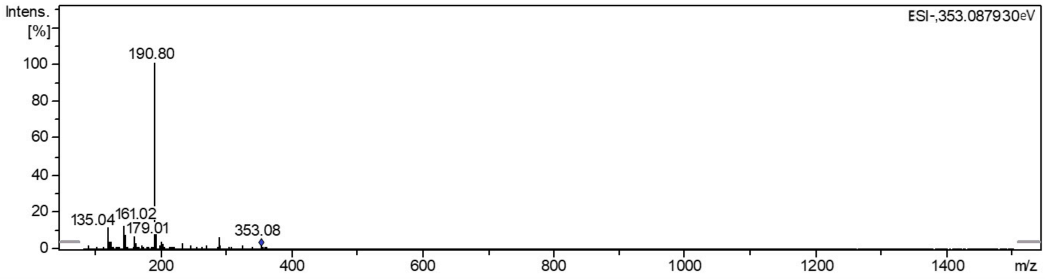


(Epi)catechin hexoside (**19**)

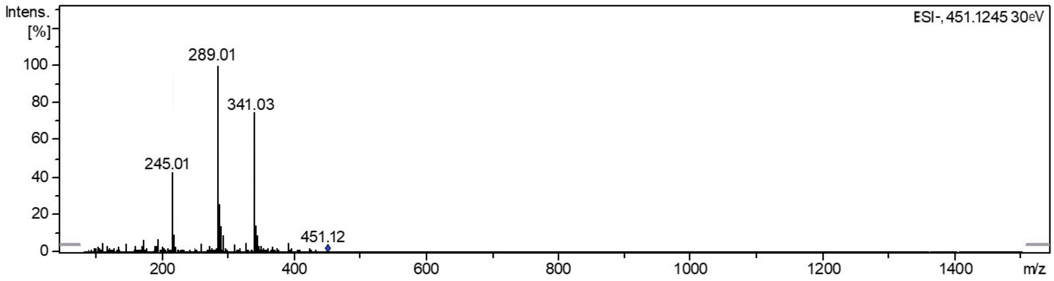


Procyanidin dimer II (**20**)

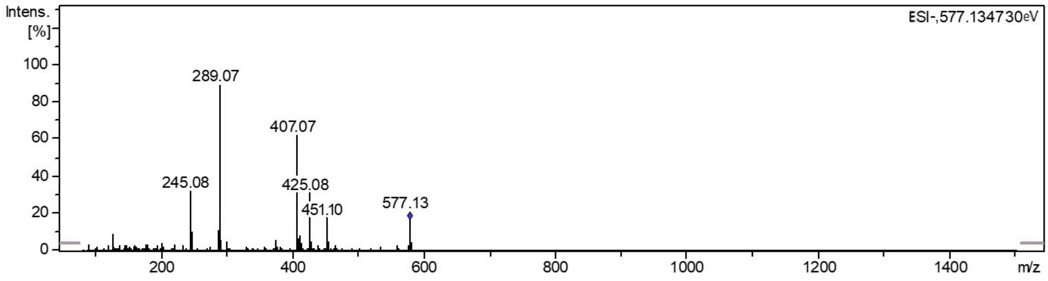


Cryptochlorogenic acid (**21**)

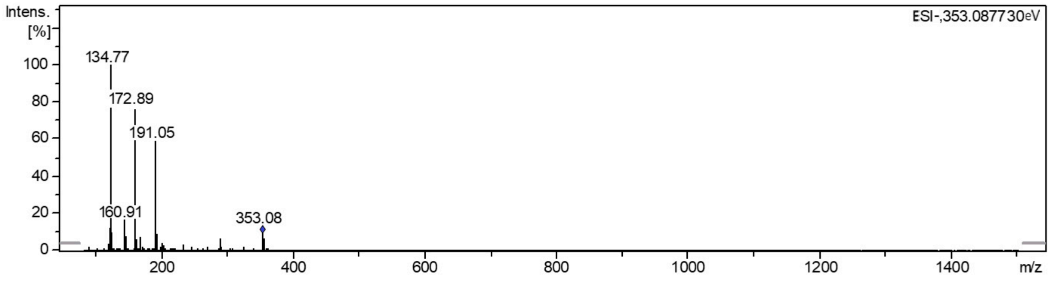

Procyanidin dimer III (**22**)

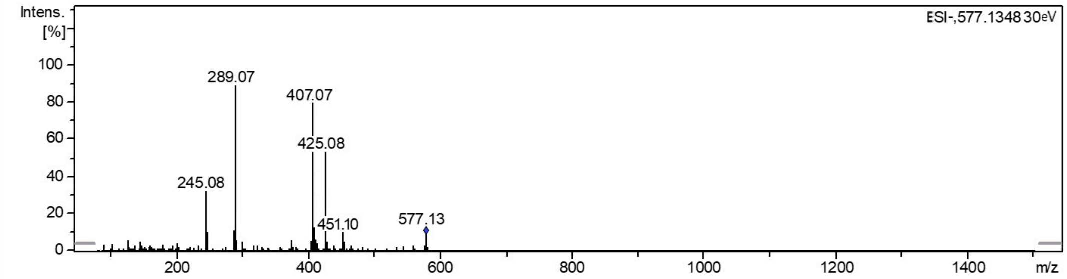


NI (**23**)

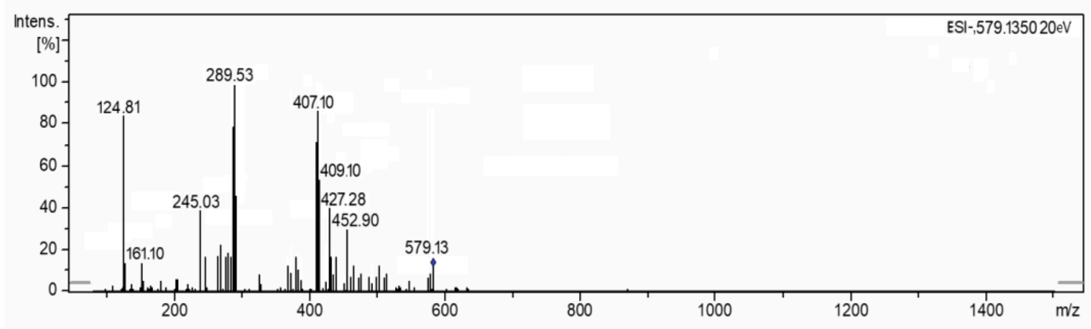


Epicatechin (**24**)

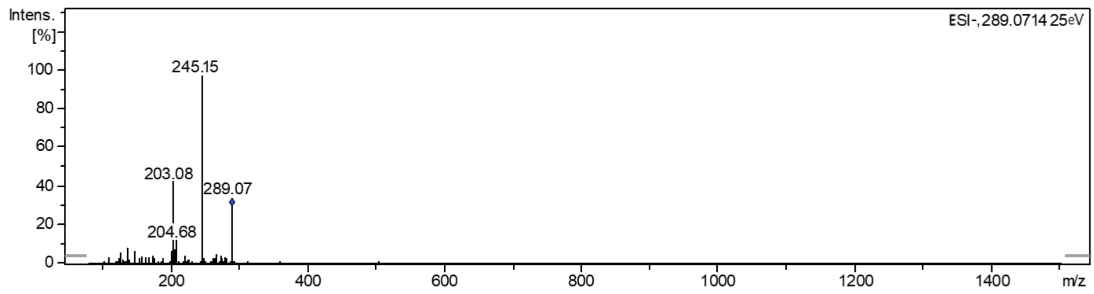


Neochlorogenic acid (**25**)

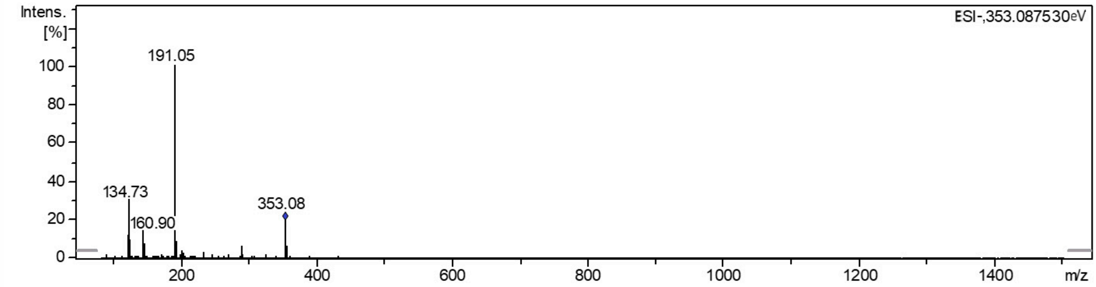


Procyanidin C1 (**27**)

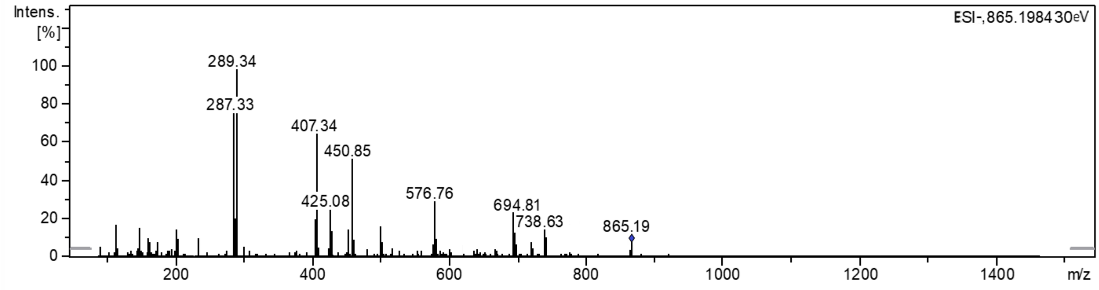

Cinchonain IIx (**28**)

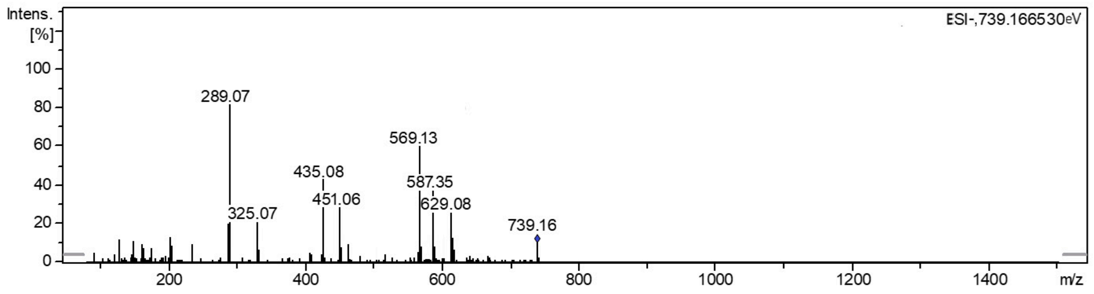


Dihydromyricetin (**29**)

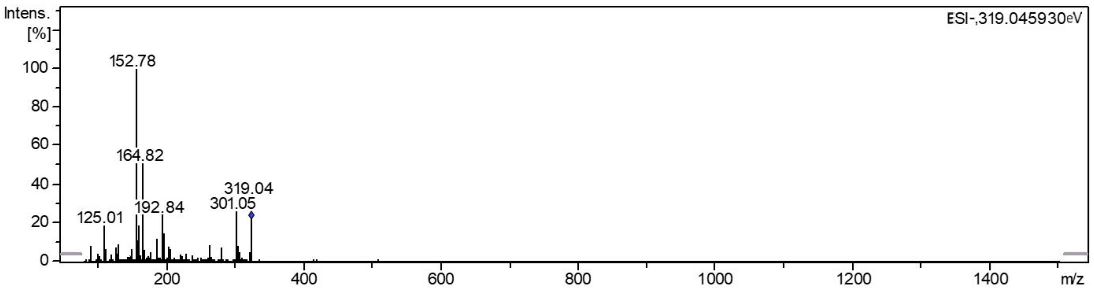


Quercetin pentoside hexoside I (**30**)

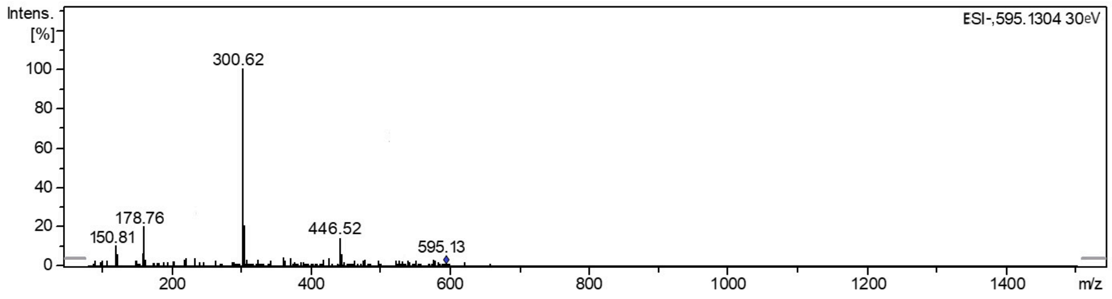


Cinchonain Ix (**31**)

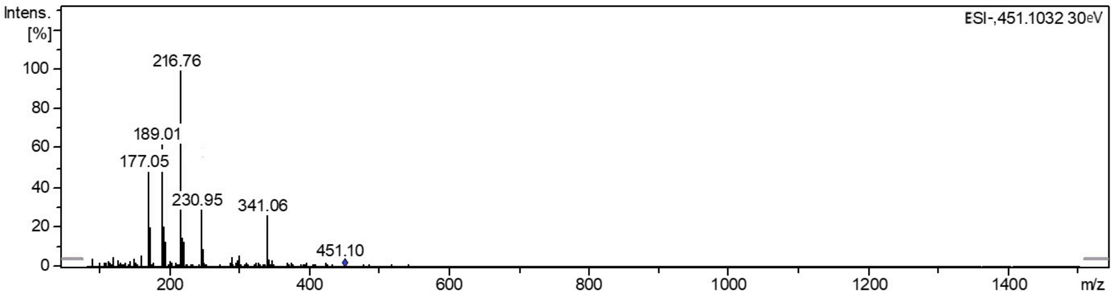


Rutin (**32**)

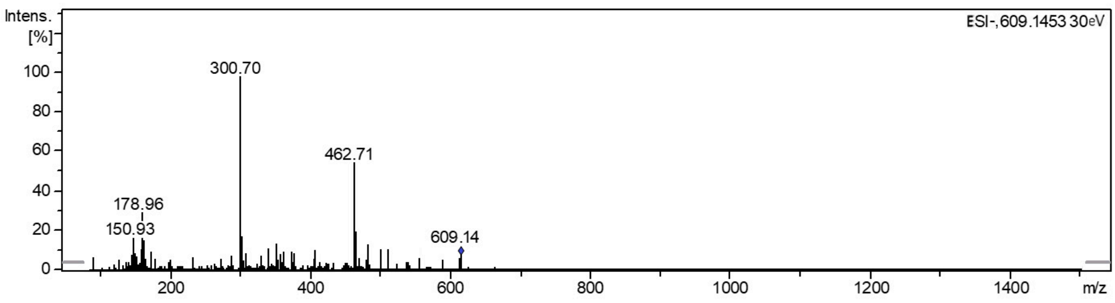

Quercetin-3-O-glucoside (**33**)

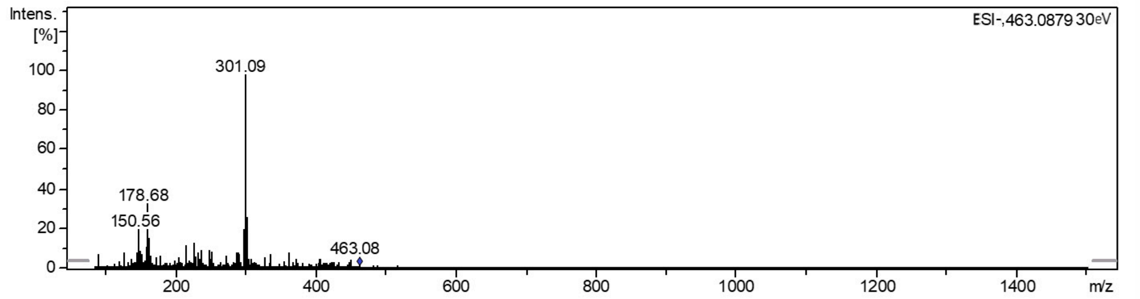


Procyanidin dimer IV (**34**)

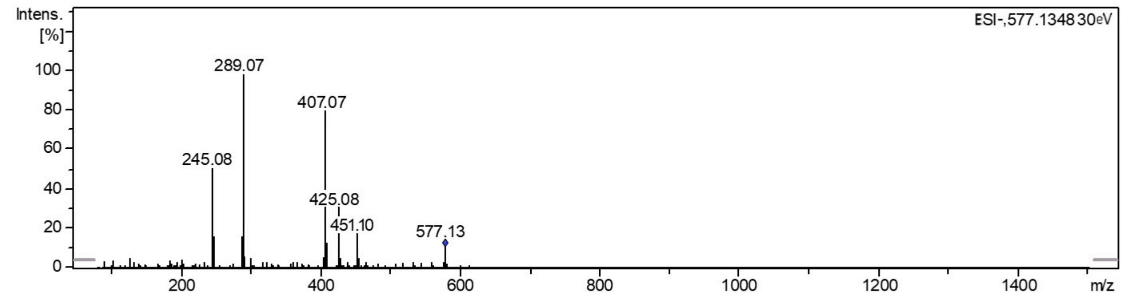


Ethylchlorogenate (**35**)

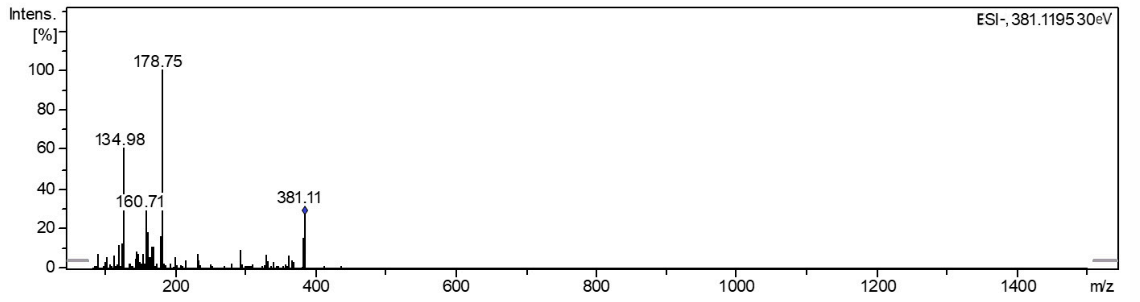


Cinchonain Ix (**36**)

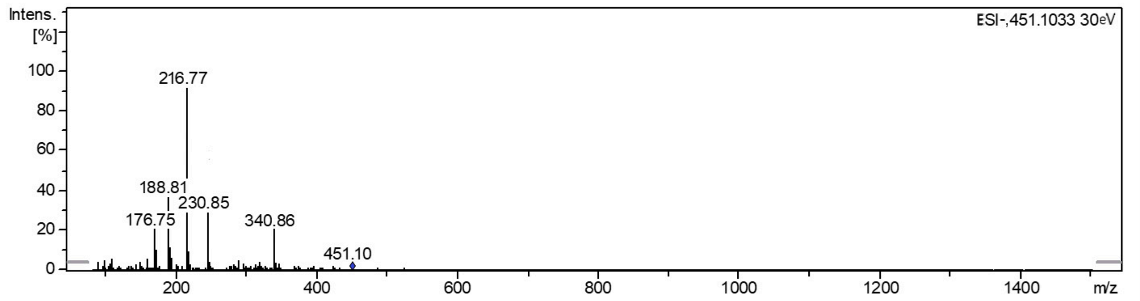


Cinchonain Ix (**37**)

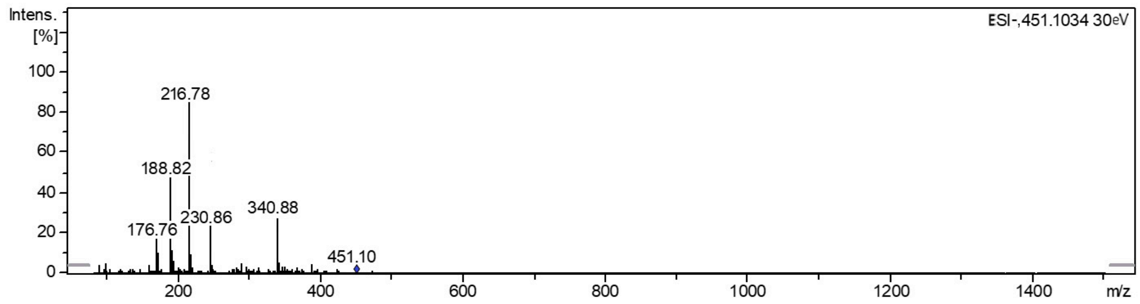

Quercetin pentoside hexoside II (**38**)

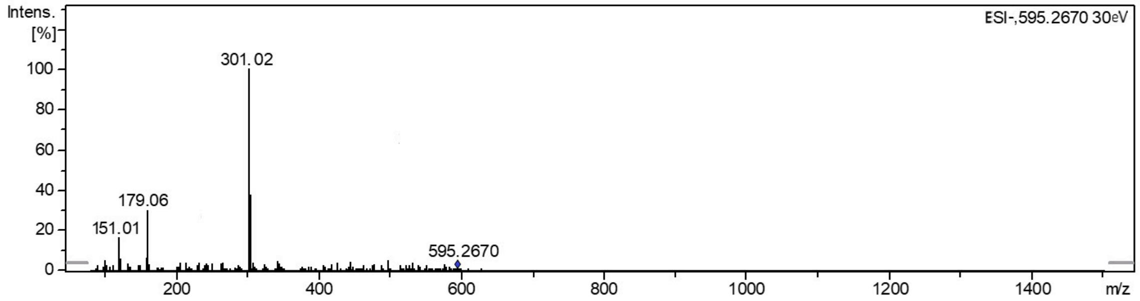


Secologanate (**39**)

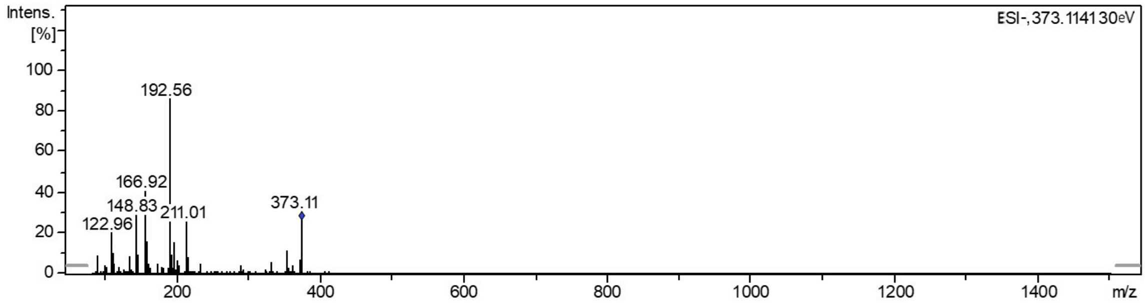


(Epi)catechin dimer monoglycoside (**40**)

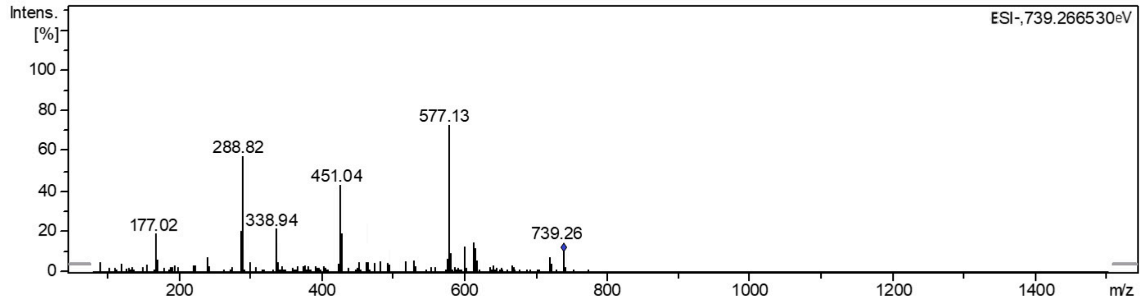


Cinchonain Ix (**41**)

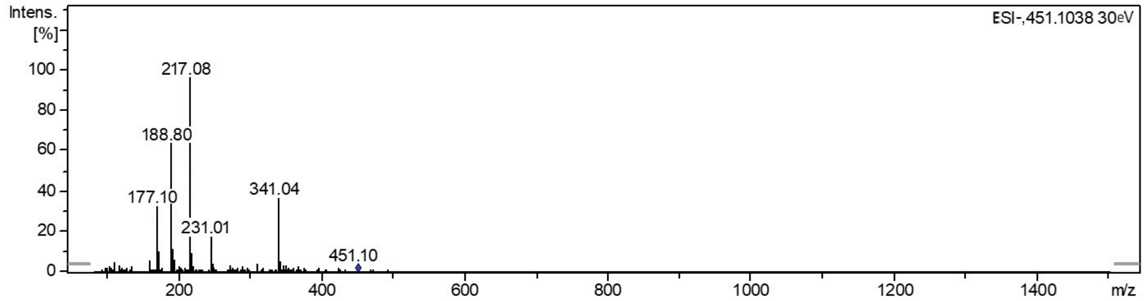


Viburtinoside derivative (**42**)

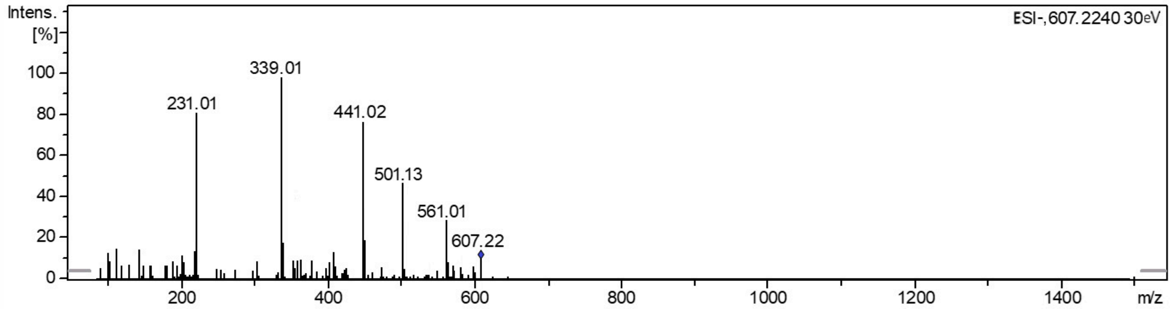

Viburtinoside derivative (**43**)

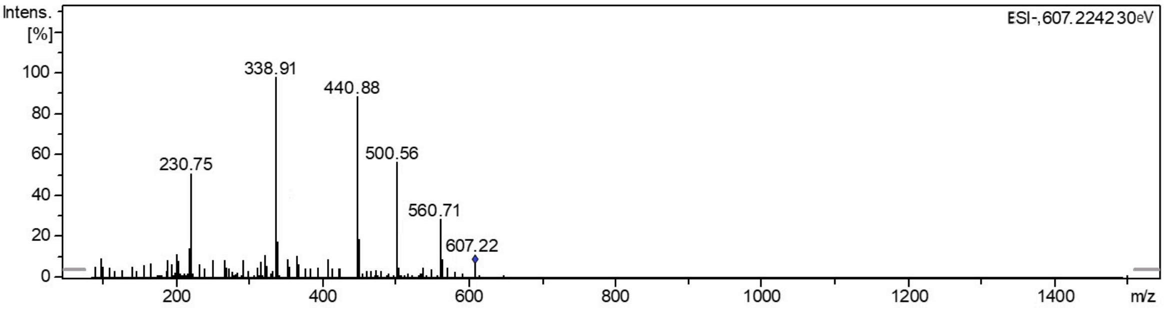


Opulus iridoid III (**44**)

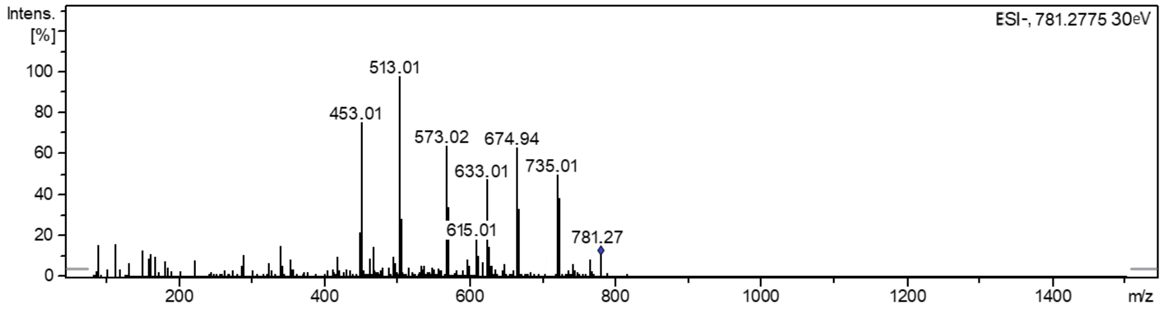


Opulus iridoid II (**45**)

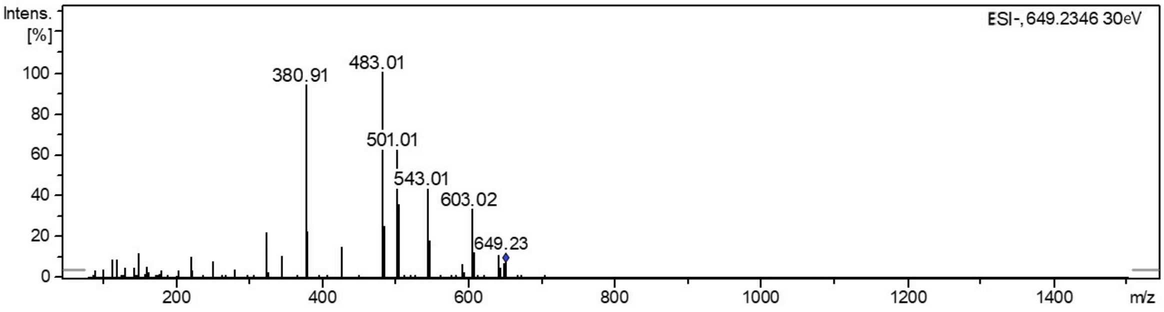

Supplement: Supplementary file 1 [file foods-09-01413-s001.pdf]
